# Standardized Regression Coefficients and Newly Proposed Estimators for ${R}^{{2}}$ in Multiply Imputed Data

**DOI:** 10.1007/s11336-020-09696-4

**Published:** 2020-03-11

**Authors:** Joost R. van Ginkel

**Affiliations:** Faculty of Social and Behavioural Sciences, Department of Methodology and Statistics, Leiden University, PO Box 9500, 2300 RB Leiden, The Netherlands

**Keywords:** missing data, multiple imputation, coefficient of determination, standardized coefficient, regression analysis

## Abstract

Whenever statistical analyses are applied to multiply imputed datasets, specific formulas are needed to combine the results into one overall analysis, also called combination rules. In the context of regression analysis, combination rules for the unstandardized regression coefficients, the *t*-tests of the regression coefficients, and the *F*-tests for testing $R^{2}$ for significance have long been established. However, there is still no general agreement on how to combine the point estimators of $R^{2}$ in multiple regression applied to multiply imputed datasets. Additionally, no combination rules for standardized regression coefficients and their confidence intervals seem to have been developed at all. In the current article, two sets of combination rules for the standardized regression coefficients and their confidence intervals are proposed, and their statistical properties are discussed. Additionally, two improved point estimators of $R^{2}$ in multiply imputed data are proposed, which in their computation use the pooled standardized regression coefficients. Simulations show that the proposed pooled standardized coefficients produce only small bias and that their 95% confidence intervals produce coverage close to the theoretical 95%. Furthermore, the simulations show that the newly proposed pooled estimates for $R^{2}$ are less biased than two earlier proposed pooled estimates.

**Electronic supplementary material:**

The online version of this article (10.1007/s11336-020-09696-4) contains supplementary material, which is available to authorized users.

## Introduction

Multiple imputation (Rubin 1987) is a widely recommended procedure to deal with missing data. The complete multiple-imputation procedure works as follows: First, for each missing value *M* values are filled in, based on a stochastic model that accurately describes the data. This results in *M* plausible complete versions of the incomplete dataset. Next, the statistical analysis of interest is applied to each of the *M* imputed datasets, resulting in *M* different outcomes of the same analysis. Finally, the *M* results are combined into one pooled statistical analysis, using specific formulas which take into account the variance of the specific parameter estimates as a result of the variance of the imputed values in the standard errors and *p*-values. These specific formulas are henceforth denoted *combination rules*.

In the specific context of regression analysis, several combination rules are available for pooling various important statistics. These combination rules involve formulas for pooling the regression coefficients (Rubin 1987), their *t*-tests (Rubin 1987), the degrees of freedom of these *t*-tests (Barnard and Rubin 1999), the *F*-test for testing $R^{2}$ for significance (Van Ginkel 2019; Rubin 1987), the *F*-test for testing $R^{2}$-change for significance (Van Ginkel 2019; Rubin 1987), and the degrees of freedom for these *F*-tests (Reiter 2007).

Although combination rules for the above-mentioned statistics in regression have been well-established, little has been written on how to combine an important statistic in regression, namely $R^{2}$. A few exceptions are Harel (2009) and Van Ginkel (2019). The small amount of literature on this topic is quite remarkable as $R^{2}$ is reported in virtually any study that uses linear regression as an indication of how well the model fits the data.

Additionally, the literature that does exist on this topic does not generally agree on what is the best way to pool $R^{2}$. Harel (2009) proposed a pooled estimate for $R^{2}$, which uses a Fisher *z* transformation. Van Ginkel (2019) criticized this method, arguing that its theoretical justification is incorrect. Van Ginkel proposed two alternatives, namely averaging $R^{2}$ across imputed datasets, and a transformation of the pooled *F*-value for testing $R^{2}$ for significance into a pooled estimate of $R^{2}$. Van Ginkel showed that despite the incorrect theoretical justification, Harel’s method still produced the best results in terms of bias, followed by averaging $R^{2}$ which produced slightly larger bias. Regarding the results of $R^{2}$, this study is rather unsatisfactory as the theoretically least sound method (Harel’s method) produced smallest bias, while the method with the best theoretical justification (transforming the *F*-value) produced the largest. Additionally, Van Ginkel pointed out a number of disadvantages of both Harel’s method and averaging $R^{2}$, which could be potential sources of bias, additional to the bias that $R^{2}$ already has in itself (e.g., Shieh 2008). In short, to date no pooled measure for $R^{2}$ in multiply imputed data seems to have been proposed that is without any theoretical objections.

Besides no general agreement on combination rules for $R^{2}$ in regression, no combination rules for the pooling of standardized regression coefficients have, to the author’s knowledge, been developed at all. However, standardized coefficients are reported in many studies that use linear regression. In fact, in many research articles standardized regression coefficients are reported instead of unstandardized regression coefficients, as standardized coefficients give an indication of the effect size of the specific predictor, independent of the scales of the variables. The fact that standardized regression coefficients are reported regularly and are occasionally even reported instead of unstandardized regression coefficients shows that there is clearly a need for combination rules for standardized regression coefficients in multiply imputed datasets.

Van Ginkel et al. (2019) stated that with a lack of a better alternative, an ad hoc method for combining standardized regression coefficients may be used, such as averaging the coefficients across the *M* imputed datasets. However, since standardized regression coefficients can be expressed as a function of the unstandardized regression coefficient, the standard deviation of the predictor, and the standard deviation of the outcome variable, there is another way to look at how the betas across multiply imputed datasets could be pooled: calculate the average of the regression coefficient across imputed datasets, calculate the average variance of the predictor and of the outcome variable across imputed datasets, and insert these quantities into the formula for the standardized regression coefficient.

Since the above-mentioned approaches are not equivalent, this firstly raises the question which approach (if any) is the correct one, and secondly, how to define “correct” in the context of multiple imputation. Normally, a convenient criterion for determining whether a parameter estimate is “correct,” is whether it is unbiased when all the assumptions for the specific analysis have been met. However, since betas are biased even in complete data (Yuan and Chan 2011), showing analytically or by means of simulations whether these approaches give unbiased estimates is not an option here. Instead, a number of other statistical properties of betas in complete data will have to be defined. Once defined, it must be verified which of these approaches (if any) has all of these statistical properties. If neither has all of the properties, the next question becomes which approach will give *the least bias* in results and bias closest to that of the same data without missing values.

In the current paper, four different conditions are defined that have to be met in complete data for a parameter estimate to be a standardized regression coefficient. It should be noted that these conditions are not based on statistical literature; they either follow from the properties of standardized coefficients directly, or have been found empirically in the author’s search for combination rules for standardized coefficients, and the mathematical proof for them was derived afterwards. For both proposed sets of combination rules, it is discussed which of these conditions they satisfy. Additionally, combination rules are proposed for the confidence intervals for these standardized regression coefficients, based on the delta method (e.g., Jones and Waller 2013).

Next, these two approaches are used for the calculation of two newly proposed pooled estimates of $R^{2}$ in multiply imputed datasets. The two proposed pooled estimates of $R^{2}$ are supposed to overcome the problems of the approaches proposed by Harel (2009) and Van Ginkel (2019). After discussing the proposed combination rules for standardized coefficient and $R^{2}$, a simulation study investigating the bias and coverage of the proposed combination rules will be discussed. Finally, a conclusion will be drawn about which combination rules for both $R^{2}$ and standardized coefficients are to be preferred.

### Pooled Estimates for Standardized Regression Coefficients in Multiply Imputed Data

Define $b_{j}$ as the unstandardized population regression coefficient of predictor ${\mathbf {x}}_{j} (j = 1,\ldots , k)$. Furthermore, define $\sigma _{{\mathbf {x}}_{j}}$ and $\sigma _{{\mathbf {y}}}$ as the population standard deviations of ${\mathbf {x}}_{j}$ and outcome variable ${\mathbf {y}}$, respectively. Lastly, define ${\varvec{\upbeta }}$ as a vector containing the standardized population regression coefficients of variables ${\mathbf {x}}_{1}\ldots {\mathbf {x}}_{k}$, which can be computed as:

1 $$\begin{aligned} {\varvec{\upbeta }}=\left[ {\begin{array}{lll} \frac{\sigma _{{\mathbf {x}}_{1}}}{\sigma _{{\mathbf {y}}}}b_{1}\\ \vdots \\ \frac{\sigma _{{\mathbf {x}}_{k}}}{\sigma _{{\mathbf {y}}}}b_{k}\\ \end{array} } \right] . \end{aligned}$$

In complete data, a sample estimate for ${\varvec{\upbeta }}$, denoted ${\hat{{\varvec{\upbeta }}}}$, can be computed as follows: Suppose that ${\hat{b}}_{j}$ is a sample estimate of the unstandardized regression coefficient of predictor variable ${\mathbf {x}}_{j}$, $s_{{\mathbf {x}}_{j}}$ is the standard deviation of ${\mathbf {x}}_{j}$, and $s_{{\mathbf {y}}}$ is the standard deviation of ${\mathbf {y}}$. The set of standardized regression coefficients in the sample is:

2 $$\begin{aligned} {\hat{{\varvec{\upbeta }}}}=\left[ {\begin{array}{c} \frac{s_{{\mathbf {x}}_{1}}}{s_{y}}{\hat{b}}_{1}\\ \vdots \\ \frac{s_{{\mathbf {x}}_{k}}}{s_{y}}{\hat{b}}_{k}\\ \end{array} } \right] . \end{aligned}$$

The first proposed set of pooled estimates for standardized regression coefficients in multiply imputed datasets is obtained by computing unbiased pooled estimates of $b_{j}$, $\sigma _{{\mathbf {x}}_{j}}^{{2}}$, and $\sigma _{{\mathbf {y}}}^{{2}}$, and substitute the estimate $b_{j}$ and the square roots of the estimates of $\sigma _{{\mathbf {x}}_{j}}^{{2}}$ and $\sigma _{{\mathbf {y}}}^{{2}}$ into Eq. 1. Because the standardization of the $b_{j}$ takes place after the pooling of $b_{j}$, $\sigma _{{\mathbf {x}}_{j}}^{{2}}$, and $\sigma _{{\mathbf {y}}}^{{2}}$, the resulting pooled beta is denoted ${\bar{{\hat{{\varvec{\upbeta }}}}}}_{\mathrm {PS}}$, where the subscript PS stands for *Pooling before Standardization*. Define ${\bar{{\hat{b}}}}_{j}=\frac{1}{M}\sum \nolimits _{m=1}^M {\hat{b}}_{j,m}, \tilde{s}_{{\mathbf {x}}_{j}}=\sqrt{\frac{1}{M}\sum \nolimits _{m=1}^M s_{{\mathbf {x}}_{j},m}^{2} }$ , and $\tilde{s}_{{\mathbf {y}}}=\sqrt{\frac{1}{M}\sum \nolimits _{m=1}^M s_{{\mathbf {y}},m}^{2} }$. ${\bar{{\hat{{\varvec{\upbeta }}}}}}_{\mathrm {PS}}$ is computed as:

3 $$\begin{aligned} {\bar{{\hat{{\varvec{\upbeta }}}}}}_{\mathrm {PS}}=\left[ {\begin{array}{c} {\bar{{\hat{{{\beta }}}}}}_{1,\,\mathrm { PS}}\\ \vdots \\ {\bar{{\hat{{{\beta }}}}}}_{ k,\, \mathrm {PS}}\\ \end{array} } \right] =\left[ {\begin{array}{c} \frac{\tilde{s}_{{\mathbf {x}}_{1}}}{\tilde{s}_{{\mathbf {y}}}} {\bar{{\hat{b}}}}_{1}\\ \vdots \\ \frac{\tilde{s}_{{\mathbf {x}}_{k}}}{\tilde{s}_{{\mathbf {y}}}} {\bar{{\hat{b}}}}_{k}\\ \end{array} } \right] . \end{aligned}$$

The second set of pooled estimates is obtained by applying Rubin’s rules for point estimates to standardized coefficients directly. In other words, average the ${\hat{{{\beta }}}}_{j,m}$’s across *M* imputed datasets, and do this for all *j*. Here, the standardization takes place before the pooling, so the resulting coefficient is denoted ${\bar{{\hat{{\varvec{\upbeta }}}}}}_{\mathrm {SP}}$, where SP denotes *Standardization before Pooling*. ${\bar{{\hat{{\varvec{\upbeta }}}}}}_{\mathrm {SP}}$ is computed as:

4 $$\begin{aligned} {\bar{{\hat{{\varvec{\upbeta }}}}}}_{\mathrm {SP}}=\left[ {\begin{array}{c} {\bar{{\hat{{{\beta }}}}}}_{\mathrm {1,SP}}\\ \vdots \\ {\bar{{\hat{{{\beta }}}}}}_{k,\, \mathrm {SP}}\\ \end{array} } \right] =\left[ {\begin{array}{c} \frac{1}{M}\sum \limits _{m=1}^M {\frac{s_{{\mathbf {x}}_{1},m}}{s_{{\mathbf {y}},m}}{\hat{b}}_{1,m}} \\ \vdots \\ \frac{1}{M}\sum \limits _{m=1}^M {\frac{s_{{\mathbf {x}}_{1},m}}{s_{{\mathbf {y}},m}}{\hat{b}}_{k,m}} \\ \end{array} } \right] . \end{aligned}$$

Next, the four conditions that can be defined for standardized regression coefficients in complete data are given. These four conditions are:

1. A standardized regression coefficient can be computed using an unbiased estimator of $b_{j}$ and estimators of $\sigma _{{\mathbf {x}}_{j}}$ and $\sigma _{{\mathbf {y}}}$ of which the squared values are unbiased estimators of $\sigma _{{\mathbf {x}}_{j}}^{{2}}$ and $\sigma _{{\mathbf {y}}}^{{2}}$, and by inserting these estimates into Eq. 1.
2. Standardized regression coefficients can also be computed by performing a regression analysis to the standardized predictors and outcome variable. The resulting regression coefficients are standardized regression coefficients.
3. A *t*-test calculated for an unstandardized regression coefficient should be insensitive to standardization of the variables. Hence, the same *t*-test calculated for the corresponding standardized regression coefficient will have the same value as of the unstandardized regression coefficient.
4. When computed for simple regression, ${\hat{{{\beta }}}}_{1}$ has the same value as the standardized regression coefficient ${\hat{{{\beta }}}}_{1,{\mathbf {y}}}$ that would be obtained if in the analysis the predictor ${\mathbf {x}}_{1}$ and outcome variable ${\mathbf {y}}$ were switched. Both ${\hat{{{\beta }}}}_{1}$ and ${\hat{{{\beta }}}}_{1,{\mathbf {y}}}$ are equal to the correlation between ${\mathbf {x}}_{1}$ and ${\mathbf {y}}$.

Regarding condition 3, it is important to note that the use of the standard *t*-test for regression coefficients would only be justified for standardized coefficients if the term $\frac{s_{{\mathbf {x}}_{1}}^{2}}{s_{{\mathbf {y}}}^{2}}$ were a fixed quantity (Jones and Waller 2013, p. 437). Since this is not the case, alternative combination rules for *t*-tests testing standardized coefficients are needed, based on the delta method (Jones and Waller 2013, pp. 439–440). Such combinations rules are proposed later on. The more general point of condition 3 is that a *t*-test should be insensitive to linear transformations, regardless of its usefulness for the transformed data.

Proof of the above four conditions in complete data is provided in Appendix I. In Appendix II, it is analytically derived which of the two sets of combination rules for the point estimates of standardized coefficients (${\bar{{\hat{{\varvec{\upbeta }}}}}}_{\mathrm {PS}}$ and ${\bar{{\hat{{\varvec{\upbeta }}}}}}_{\mathrm {SP}}$) meet which of the four conditions. Table 1 gives a summary of the analytical results in Appendix II.

**Table 1:** Overview of which set of combination rules (${\bar{{\hat{{\varvec{\beta }}}}}}_\mathrm{PS}\,$ and ${\bar{{\hat{{\varvec{\beta }}}}}}_\mathrm{SP}$) satisfy which of the four conditions of standardized regression coefficients

Condition	Combination rule	
	${\bar{{\hat{{\varvec{\upbeta }}}}}}_{\mathrm {PS}}$	${\bar{{\hat{{\varvec{\upbeta }}}}}}_{\mathrm {SP}}$
1. ${\hat{{{\beta }}}}_{j}$ can be computed using unbiased estimators of $b_{j}$, $\sigma _{x_{j}}^{2}$, and $\sigma _{y}^{2}$.	Yes	No
2. ${\hat{{{\beta }}}}_{j}$ can be computed by performing regression to standardized ${\mathbf {X}}$ and ${\mathbf {y}}$	Yes	Yes
3. *t*-test calculated for ${\hat{b}}_{j}$ has same value as same *t*-test calculated for ${\hat{{{\beta }}}}_{j}$	Yes	No
4. In simple regression, ${\hat{{{\beta }}}}_{1}$, ${\hat{{{\beta }}}}_{1,y}$, and $r_{x_{1}y}$ are the same.	No	Yes

### Statistical Tests for Standardized Regression Coefficients in Multiply Imputed Data

As noted earlier, *t*-tests for standardized regression coefficients cannot be used for the standardized regression coefficients because the estimates of the variances of ${\mathbf {X}}$ and ${\mathbf {y}}$ are not fixed quantities. For complete data, Jones and Waller (2013, p. 440; also, see Yuan and Chan 2011) discuss a *t*-test for ${\hat{{{\beta }}}}_{j}$ using a standard error based on the delta method. Define $\hat{c}_{j}$ as the *j*th diagonal element of the covariance matrix of the predictors, ${\mathbf {S}}_{{\mathbf {X}}}$. This standard error is computed as:

5 $$\begin{aligned} SE\left( {\hat{{{\beta }}}}_{j} \right) =\sqrt{\frac{s_{{\mathbf {x}}_{j}}^{2} \hat{c}_{j}s_{e}^{2}}{(n-3)s_{{\mathbf {y}}}^{2}}+\frac{{\hat{b}}_{j}^{2}\left[ s_{{\mathbf {x}}_{j}}^{2}\left( {\hat{\mathbf {b'}}}{{\mathbf {S}}}_{{\mathbf {X}}}{\hat{\mathbf {b}}} \right) -s_{{\mathbf {x}}_{j}}^{2}s_{e}^{2}-s_{{\mathbf {x}}_{j}{\mathbf {y}}}^{2} \right] }{(n-3)s_{{\mathbf {y}}}^{4}}}. \end{aligned}$$

For multiply imputed data, define $\tilde{s}_{e}=\sqrt{\frac{1}{M}\sum \nolimits _{m=1}^M s_{e,m}^{2} }$, $\tilde{c}_{j}=\sqrt{\frac{1}{M}\sum \nolimits _{m=1}^M \hat{c}_{j,m} }$, $\tilde{s}_{{\mathbf {x}}_{j}{\mathbf {y}}}=\frac{1}{M}\sum \nolimits _{m=1}^M s_{{\mathbf {x}}_{j}{\mathbf {y}}}$, and ${\bar{\mathbf {S}}}_{{\mathbf {X}}}=\frac{1}{M}\sum \nolimits _{m=1}^M {\mathbf {S}}_{{\mathbf {X}},m}$. Applying Rubin’s rules for standard errors, and extending the principle of Pooling before Standardization, the proposed within-imputation variance, between-imputation variance, and total variance of ${\bar{{\hat{{{\beta }}}}}}_{j\mathrm {,PS}}$ are:

$$\begin{aligned} \bar{U}_{\mathrm {PS}}= & {} \frac{\tilde{s}_{{\mathbf {x}}_{j}}^{2} \tilde{c}_{j}\tilde{s}_{e}^{2}}{(n-3)\tilde{s}_{{\mathbf {y}}}^{2}} +\frac{{\bar{{\hat{b}}}}_{j}^{2}\left[ \tilde{s}_{{\mathbf {x}}_{j}}^{2}\left( {\bar{{\hat{\mathbf {b'}}}}}{{\bar{\mathbf {S}}}}_{{\mathbf {X}}} {\bar{{\hat{\mathbf {b}}}}} \right) -\tilde{s}_{{\mathbf {x}}_{j}}^{2}\tilde{s}_{e}^{2}-s_{{\mathbf {x}}_{j}{\mathbf {y}}}^{2} \right] }{(n-3)\tilde{s}_{{\mathbf {y}}}^{4}}, \\ B_{\mathrm {PS}}= & {} \frac{\tilde{s}_{{\mathbf {x}}_{j}}^{2}}{\tilde{s}_{{\mathbf {y}}}^{2}(M-1)}\sum \limits _{m=1}^M {({\hat{b}}_{j,m}-{\bar{{\hat{b}}}}_{j})}^{2} =\frac{\tilde{s}_{{\mathbf {x}}_{j}}^{2}}{\tilde{s}_{{\mathbf {y}}}^{2}}Var({\hat{b}}_{j,m}), \\ T_{\mathrm {PS}}= & {} \bar{U}_{\mathrm {PS}}+\left( 1+M^{-1} \right) B_{\mathrm {PS}}={SE}_{\mathrm {PS}}^{2}. \end{aligned}$$

The corresponding *t*-value testing ${\bar{{\hat{{{\beta }}}}}}_{j\mathrm {,PS}}$ for significance is:

$$\begin{aligned} t=\frac{{\bar{{\hat{{{\beta }}}}}}_{j\mathrm {,PS}}}{{SE}_{\mathrm {PS}}}, \end{aligned}$$

and the ($1-\alpha$)% confidence interval is:

$$\begin{aligned} {\bar{{\hat{{{\beta }}}}}}_{j\mathrm {,PS}} \,\pm \, t_{1-\alpha /2,df}{SE}_{\mathrm {PS}}, \end{aligned}$$

where *df* is approximated using an approximation from Barnard and Rubin (1999).

Applying Rubin’s rules for standard errors to the standard errors of ${\bar{{\hat{{{\beta }}}}}}_{j\mathrm {,SP}}$, the within-imputation variance, between-imputation variance, and total variance of ${\bar{{\hat{{{\beta }}}}}}_{j\mathrm {,SP}}$ become:

$$\begin{aligned} \bar{U}_{\mathrm {SP}}= & {} \frac{1}{M}\sum \limits _{m=1}^M {SE\left( {\hat{{{\beta }}}}_{j,m} \right) },\\ B_{\mathrm {SP}}= & {} \frac{1}{(M-1)}\sum \limits _{m=1}^M {({\hat{{{\beta }}}}_{j,m} -{\bar{{\hat{\beta }}}}_{j})}^{2} =Var({\hat{{{\beta }}}}_{j}),\\ T_{\mathrm {SP}}= & {} \bar{U}_{\mathrm {SP}}+\left( 1+M^{-1} \right) B_{\mathrm {SP}}={SE}_{\mathrm {SP}}^{2}. \end{aligned}$$

The corresponding *t*-value testing ${\bar{{\hat{\beta }}}}_{j\mathrm {,SP}}$ for significance is:

$$\begin{aligned} t=\frac{{\bar{{\hat{\beta }}}}_{j\mathrm {,SP}}}{{SE}_{\mathrm {SP}}}, \end{aligned}$$

and the ($1-\alpha$)% confidence interval is:

$$\begin{aligned} {\bar{{\hat{\beta }}}}_{j\mathrm {,SP}}{\,\pm \,}t_{1-\alpha /2,df}{SE}_{\mathrm {SP}}. \end{aligned}$$

Of the two above-described methods, the confidence interval and statistical test for ${\bar{{\hat{\beta }}}}_{j\mathrm {,SP}}$ have a better theoretical justification because the combination rules for ${\bar{{\hat{\beta }}}}_{j\mathrm {,SP}}$ as defined above are simply Rubin’s combination rules applied to the ${\hat{{{\beta }}}}_{j,m}\mathrm{'s}$ directly, without any modification. When in complete data the sampling distribution of ${\hat{{{\beta }}}}_{j}$ is (approximately) normal with the standard error as in Eq. 5, and in the incomplete-data case the imputation method is Bayesianly proper (Schafer 1997, p. 105) in principle, all conditions for applying Rubin’s rules (1987, Chap. 3) have been met. In the next subsection, combination rules for $R^{2}$ that use ${\bar{{\hat{{\varvec{\upbeta }}}}}}_{\mathrm {PS}}$ and ${\bar{{\hat{{\varvec{\upbeta }}}}}}_{\mathrm {SP}}$ are derived.

### Pooled Estimates for $R^{2}$ in Multiply Imputed Data

Thus far, three different point estimates for $R^{2}$ for multiple regression in multiply imputed data have been proposed. Harel (2009) proposed a pooled estimate based on a Fisher z transformation. This procedure works as follows: First, a Fisher z transformation is carried out to the square root of each $R_{m}^{2}$ ($m = 1,\ldots , M$). Next, the Fisher z transformations are averaged across the *M* imputed datasets. Finally, the average Fisher z-transformed square root of $R^{2}$ is transformed back using an inverse Fisher z transformation, and squaring the result. The resulting pooled estimate is denoted ${\mathfrak {R}}^{2}$.

Van Ginkel (2019) criticized Harel’s approach, arguing that the use of a Fisher z transformation is based on an incorrect justification. Instead, he proposed two alternative pooled estimates for $R^{2}$: The average of the $R_{m}^{2}\mathrm{'s}$ across imputed datasets, denoted $\bar{R^{2}}$, and an estimate that is computed from the pooled *F*-value for testing $R^{2}$ for significance, denoted $R_{F}^{2}$. In a simulation study, Van Ginkel (2019) showed that $R_{F}^{2}$ was substantially lower than the $R^{2}$ of the same data without missing values and was thus not recommended. $\bar{R^{2}}$ and ${\mathfrak {R}}^{2}$, on the other hand, were somewhat more positively biased than the $R^{2}$ of the same data without missing values. This overestimation was somewhat larger for $\bar{R^{2}}$ than for ${\mathfrak {R}}^{2}$.

Although both $\bar{R^{2}}$ and ${\mathfrak {R}}^{2}$ gave substantially better estimates of $R^{2}$ than $R_{F}^{2}$, Van Ginkel (2019) still pointed out a potential problem of both methods. When $\rho ^{2}$ is close to zero, both $\bar{R^{2}}$ and ${\mathfrak {R}}^{2}$ will be higher than $R^{2}$ in case of no missing data. This phenomenon can best be demonstrated using the bivariate case with only one predictor. When there is hardly a relationship between ${\mathbf {x}}_{1}$ and ${\mathbf {y}}$, it may happen that in some imputed datasets the correlation between ${\mathbf {x}}_{1}$ and ${\mathbf {y}}$ is slightly negative and in other imputed datasets slightly positive. Since in simple regression $R^{2}$ equals the squared correlation between both variables, averaging $R_{m}^{2}\mathrm{'s}$ will ignore the sign differences in correlations across imputed dataset. Consequently, $\bar{R^{2}}$ will be higher than the square of the average correlation across imputed datasets. ${\mathfrak {R}}^{2}$ has the same problem because it also ignores sign differences between correlations across imputed datasets.

In the multiple-regression case, something similar may happen when in one imputed dataset the regression coefficient of variable ${\mathbf {x}}_{j}$ is slightly negative and in another imputed dataset slightly positive. If the averaging takes place at the level of (a transformation of) $R^{2}$ rather than at the level of the individual regression coefficient in coming to a pooled estimate of $R^{2}$, predictors with almost zero relation with the outcome variable will give a larger contribution to the pooled estimate of $R^{2}$ than they are supposed to give.

For simple regression, the problem may be resolved by averaging the correlation across imputed datasets, and taking the square of the average. Unfortunately, this cannot be done in multiple regression because in multiple regression the only correlation that can be squared to arrive at $R^{2}$ is the correlation between the observed and predicted values of ${\mathbf {y}}$, which is always positive (in simple regression, this correlation equals the absolute value of the correlation between ${\mathbf {x}}_{1}$ and ${\mathbf {y}}$). However, we can formulate a generalization of squaring the average correlation to multiple regression by making use of the fact that in complete data, $R^{2}$ may also be expressed as:

6 $$\begin{aligned} R^{2}=\sum \limits _{j=1}^k r_{{\mathbf {x}}_{j}{\mathbf {y }}} {\hat{{{\beta }}}}_{j}\mathrm {.} \end{aligned}$$

By filling in pooled estimates for $r_{{\mathbf {x}}_{j}{\mathbf {y}}}$ and ${\hat{{{\beta }}}}_{j}$, we can come at a pooled estimate of $R^{2}$ in multiply imputed data, in which variables with weak relations with the outcome variable will not have a disproportionally large contribution. Using the two sets of pooled estimates for standardized regression coefficients that were previously defined, two pooled versions of $R^{2}$ are proposed. The first pooled estimate uses the elements in ${\bar{{\hat{{\varvec{\upbeta }}}}}}_{\mathrm {PS}}$ and the pooled standard deviations $\tilde{s}_{{\mathbf {x}}_{j}}$ and $\tilde{s}_{{\mathbf {y}}}$. As already mentioned, in complete data and simple regression the standardized coefficient and the correlation between the predictor and the outcome are equal. Making use of this fact, pooled estimates of the correlations between the predictors and the outcome are computed by carrying out simple regressions to the *M* imputed datasets with ${\mathbf {y}}$ as the outcome variable, for each predictor ${\mathbf {x}}_{j}$. For each variable, a pooled standardized simple regression coefficient is obtained using the method of ${\bar{{\hat{{\varvec{\upbeta }}}}}}_{\mathrm {PS}}$. This estimate is denoted $r_{{\mathbf {x}}_{j}{\mathbf {y}},{\mathrm {PS}}}$.

As a pooled estimate for ${\hat{{{\beta }}}}_{j}$ in Eq. 6, we use ${\bar{{\hat{\beta }}}}_{j,\mathrm {PS}}$. The resulting pooled estimate for $R^{2}$ is:

$$\begin{aligned} \hat{R}_{\mathrm {PS}}^{{2}}=\sum \limits _{j=1}^k r_{{\mathbf {x}}_{j}{\mathbf {y},{\mathrm {PS}}}} {\bar{{\hat{\beta }}}}_{j,\mathrm {PS}}\mathrm {.} \end{aligned}$$

Although it is shown in Table 1 (condition 4) that in simple regression ${\bar{{\hat{\beta }}}}_{1,\mathrm {PS}}$ is not equal to the average correlation between ${\mathbf {x}}_{1}$ and ${\mathbf {y}}$, it was still decided to use $r_{{\mathbf {x}}_{j}{\mathbf {y},{\mathrm {PS}}}}$ (which equals ${\bar{{\hat{\beta }}}}_{1,\mathrm {PS}}$ in a simple regression of ${\mathbf {x}}_{j}$ on ${\mathbf {y}}$) as an estimate of the correlation in $\hat{R}_{\mathrm {PS}}^{{2}}$. The reason is that if the average correlation across imputed datasets were used, the resulting pooled $R^{2}$ would not be equal to the square of the pooled standardized regression coefficient anymore, in case of simple regression.

The second pooled estimate of $R^{2}$ is based on ${\bar{{\hat{{\varvec{\upbeta }}}}}}_{\mathrm {SP}}$. Define $\bar{r}_{{\mathbf {x}}_{j}{\mathbf {y}}}$ as the average correlation between ${\mathbf {x}}_{j}$ and ${\mathbf {y}}$ across imputed datasets (which is, in a simple regression of ${\mathbf {x}}_{j}$ on ${\mathbf {y}}$, equal to ${\bar{{\hat{\beta }}}}_{1,\mathrm {SP}}$). Substituting $\bar{r}_{{\mathbf {x}}_{j}{\mathbf {y}}}$ for $r_{{\mathbf {x}}_{j}{\mathbf {y}}}$ in Eq. 6, and ${\bar{{\hat{\beta }}}}_{j,\mathrm {SP}}$ obtained from the multiple regression of ${\mathbf {X}}$ on ${\mathbf {y}}$ for ${\hat{{\beta }}}_{j}$, we get:

$$\begin{aligned} \hat{R}_{\mathrm {SP}}^{{2}}=\sum \limits _{j=1}^k \bar{r}_{{\mathbf {x}}_{j}{\mathbf {y}}} {\bar{{\hat{{{\beta }}}}}}_{j,\mathrm {SP}}{.} \end{aligned}$$

### Evaluating Advantages and Disadvantages of each Approach

An advantage of $\hat{R}_{\mathrm {SP}}^{{2}}$ over $\hat{R}_{\mathrm {PS}}^{{2}}$ is that in simple regression, $\hat{R}_{\mathrm {SP}}$, $\vert \bar{r}_{{\mathbf {x}}_{1}{\mathbf {y}}}\vert$, and $\vert {\bar{{\hat{\beta }}}}_{1\mathrm {,SP}}\vert$, are all equal; just like in complete data, *R*, $\vert r_{{\mathbf {x}}_{j}{\mathbf {y}}}\vert$, and $\vert {\hat{{{\beta }}}}_{1}\vert$ are equal (condition 4, Table 1). The same is not true for $\hat{R}_{\mathrm {PS}}^{{2}}$, of which the square root in case of simple regression will not equal $\vert r_{{\mathbf {x}}_{j}{\mathbf {y},{\mathrm {PS}}}}\vert$. This also illustrates an important advantage of ${\bar{{\hat{{\varvec{\upbeta }}}}}}_{\mathrm {SP}}$ over ${\bar{{\hat{{\varvec{\upbeta }}}}}}_{\mathrm {PS}}$ because the elements in ${\bar{{\hat{{\varvec{\upbeta }}}}}}_{\mathrm {SP}}$ are used for calculating $\hat{R}_{\mathrm {SP}}^{{2}}.$ Thus, when in a multiply imputed dataset a simple regression is carried out, the values of $\hat{R}_{\mathrm {SP}}^{{2}}$, $\bar{r}_{{\mathbf {x}}_{1}{\mathbf {y}}}\mathrm {,}\, {\bar{{\hat{{{\beta }}}}}}_{1,\mathrm {SP}}$, and even ${\bar{{\hat{{{\beta }}}}}}_{\mathrm {1,y,SP}}$ all relate to one another the way they are supposed to be related.

A disadvantage of ${\bar{{\hat{{\varvec{\upbeta }}}}}}_{\mathrm {SP}}$ is that it violates condition 3 (Table 1). This is an advantage of ${\bar{{\hat{{\varvec{\upbeta }}}}}}_{\mathrm {PS}}$ over ${\bar{{\hat{{\varvec{\upbeta }}}}}}_{\mathrm {SP}}$. However, the question is to what extent this violation of condition 3 is problematic. Given that standardized regression coefficients are tested using a different *t*-test anyway, this violation of assumption might as well simply be ignored.

Since all proposed pooled estimates of $R^{2}$ and ${\varvec{\upbeta }}$ have their disadvantages, and none of them meet all of the necessary conditions, an important question becomes how well they perform in terms of bias and coverage. If one method clearly produces a less biased estimate of the population parameter in question and the population parameter is better covered by the confidence interval than the other, the method producing the least bias and best coverage is the preferred one. To answer the question about bias and coverage, all methods discussed here were investigated in a simulation study, which is discussed next.

## Method

### Fixed Design Characteristics

The general form of the simulation model in this study was:

7 $$\begin{aligned} y_{i}=b_{0}+b_{1}x_{1i}+b_{2}x_{2i}+b_{3}x_{3i}+b_{4}x_{4i}+\varepsilon _{i} \end{aligned}$$

(a regression model with four predictors). The values of $b_{0}$ to $b_{4}$ were varied and are discussed later on in the independent variables section. The means of predictors ${\mathbf {x}}_{1}$ to ${\mathbf {x}}_{4}$ were ${{\varvec{{\upmu }}}} =(\mu _{1},\mu _{2},\mu _{3},\mu _{4}) = (2,5,10,20)$. The covariance matrix for the predictors is:Values of ${\varvec{\upmu }}$ and ${\varvec{\Sigma }}$ were based on earlier studies by Harel (2009) and Van Ginkel (2019). The variance of the error term $\varepsilon _{i}$ was set at $\sigma _{\varepsilon }^{2}=0.6$. Using these means and covariances, in each design cell, one thousand ($D = 1000$) replicated sets of predictors were drawn from a specific population distribution and with a specific sample size (both to be discussed in the independent variables section). Additionally, for each case in the sample, an $\varepsilon _{i}$ was drawn from $N(0,\, \sigma _{\varepsilon }^{2})$. In each replicated dataset, ${\mathbf {y}}$ was constructed using the model in Eq. 7.

Next, the complete datasets were made incomplete with a specific percentage of missingness, and using a specific missingness mechanism. The resulting incomplete datasets were multiply imputed using fully conditional specification (FCS; Van Buuren 2012, p. 108–116; Van Buuren et al. 2006) using the mice package (Van Buuren and Groothuis-Oudshoorn 2011) in R (R Development Core Team 2018). The number of imputations was fixed at $M = 25$.

### Independent Variables

*Value of *$\rho ^{2}$ Two values of $\rho ^{2}$ were studied. Their values and the values of their corresponding regression coefficients were:

$$\begin{aligned} \rho ^{2}= & {} 0 \qquad \qquad \qquad \qquad \rho ^{2}=0.455\\ {\mathbf {b}}= & {} \left[ {\begin{array}{c} b_{0}\\ b_{1}\\ b_{2}\\ b_{3}\\ b_{4}\\ \end{array} } \right] =\left[ {\begin{array}{c} 0\\ 0\\ 0\\ 0\\ 0\\ \end{array} } \right] \quad {\mathbf {b}}=\left[ {\begin{array}{c} b_{0}\\ b_{1}\\ b_{2}\\ b_{3}\\ b_{4}\\ \end{array} } \right] =\left[ {\begin{array}{c} 0.2\\ 0.1\\ 0.1\\ 0.2\\ 0.1\\ \end{array} } \right] . \end{aligned}$$

The corresponding standardized regression coefficients (computed using Eq. 2) were:

$$\begin{aligned} \rho ^{2}= & {} 0 \qquad \qquad \qquad \qquad \rho ^{2}=0.455\\ {\varvec{\upbeta }}= & {} \left[ {\begin{array}{c} \beta _{1}\\ \beta _{2}\\ \beta _{3}\\ \beta _{4}\\ \end{array} } \right] =\left[ {\begin{array}{c} 0\\ 0\\ 0\\ 0\\ \end{array} } \right] \quad {\varvec{\upbeta }}=\left[ {\begin{array}{c} \beta _{1}\\ \beta _{2}\\ \beta _{3}\\ \beta _{4}\\ \end{array} } \right] =\left[ {\begin{array}{c} 0.213\\ 0.213\\ 0.426\\ 0.302\\ \end{array} } \right] . \end{aligned}$$

*Sample size* Two different sample sizes were studied: $N = 100$ and $N = 500$.

*Population distribution* To see how robust the proposed combination rules for standardized regression coefficients and $R^{2}$ were to different types of population distributions, the predictors were simulated under a multivariate normal distribution and under a multivariate lognormal distribution with the same means and covariances. Skewness and kurtosis of predictors ${\mathbf {x}}_{1}$ to ${\mathbf {x}}_{4}$ were (4.75, 1.43, 0.68, 0.48) and (57.60, 3.85, 0.84, 0.41), respectively. Random values from a multivariate normal distribution were generated using the mvnorm procedure in the MASS package (Venables and Ripley 2002) in R; random values from a lognormal distribution were generated using the mvlognormal function in the MethylCapSig package (Ayyala et al. 2016).

Within the mice package, there are two different variants of FCS, namely the regression approach (e.g., Van Buuren 2012, p. 13; Little and Schenker 1995, p. 60) and predictive mean matching (Van Buuren 2012, p. 68–74; Van Buuren et al. 2006; Rubin 1986). The regression approach imputes each variable using a normal regression model with the other variables as predictors for the missing data. When the data are multivariate normally distributed, this is the preferred method for imputation. However, when the data are not normally distributed (such as in case of a multivariate lognormal distribution), the regression approach may not preserve the shapes of the distributions of the variables. Predictive mean matching is a method that has been shown to better preserve distributional shapes when data are not normally distributed than the regression approach (Marshall et al. 2010a; Marshall et al. 2010b). Thus, for the current simulation study it was decided to impute the data using the regression approach when the data were normally distributed, and to use predictive mean matching when the data were multivariate lognormally distributed.

*Percentage of missingness* In each simulated complete dataset, missingness was simulated with three different percentages of missing data: 12.5%, 25%, and 50%. These percentages were based on Van Ginkel (2019).

*Missingness mechanism* Missingness was simulated according to two missingness mechanisms, namely *missing completely at random* (MCAR) and missing at random (MAR; Little and Rubin 2002, p. 10). Under MCAR, variable ${\mathbf {x}}_{1}$ was always observed; for the remaining variables ${\mathbf {x}}_{2}$ to ${\mathbf {y}}$, the following strategy was adopted: An $N \times k$ matrix **A **with uniform random numbers ranging from 0 to 1 was generated. For a specific proportion of missingness *c*, the $c \times N \times k$ highest entries in matrix **A** were removed in matrix [${\mathbf {x}}_{2},\,{\mathbf {x}}_{3},\, {\mathbf {x}}_{4}{\mathbf {,\, y}}$].

Under MAR, besides matrix **A **the following matrix ${\mathbf {W}}={\mathbf {x}}_{1}{\mathbf {1}}_{k}^{\mathbf {'}} -[\mathrm {min}\mathbf {(}{\mathbf {x}}_{1})- 0.01]\times \mathbf {1}_{\mathbf {N}}{\mathbf {1}}_{k}^{\mathbf {'}}$ was computed. The $c \times N \times k$ highest entries in matrix ${\mathbf {W*A}}$ were removed in matrix [${\mathbf {x}}_{2},\,{\mathbf {x}}_{3},\, {\mathbf {x}}_{3},\,{\mathbf { y}}$]. Thus, the higher the value of ${\mathbf {x}}_{1}$, the higher the probability of missing data on the other variables.

*Set of combination rules* The influence of two different sets combination rules on the estimates of the standardized regression coefficients and their confidence intervals was studied, namely ${\bar{{\hat{{\varvec{\upbeta }}}}}}_{\mathrm {PS}}$ and ${\bar{{\hat{{\varvec{\upbeta }}}}}}_{\mathrm {SP}}$. For estimates of $R^{2}$, the influence of four different combination rules was studied: the two newly proposed pooled estimates, $\hat{R}_{\mathrm {PS}}^{{2}}$ and $\hat{R}_{\mathrm {SP}}^{{2}}$, and two existing methods, namely ${\mathfrak {R}}^{2}$ (Harel 2009), and $\bar{R^{2}}$ (Van Ginkel 2019). Methods ${\mathfrak {R}}^{2}$ and $\bar{R^{2}}$ were included to serve as a benchmark for comparing the new methods with. The development of $\hat{R}_{\mathrm {PS}}^{{2}}$ and $\hat{R}_{\mathrm {SP}}^{{2}}$ as pooled estimates of $R^{2}$ can be considered successful when they produce less bias than the existing simple method that lacks a theoretical justification ($\bar{R^{2}}$) and produce at least as little bias as (but preferably less bias than) the existing method that works better than $\bar{R^{2}}$, but has an incorrect justification (${\mathfrak {R}}^{2}$).

**Table 2:** Bias (standard deviations between brackets) and coverage percentages of the estimated $\beta _{j}$s, and bias in estimates of $\rho ^{2}$

*N*	Missingness mechanism	$\hat{Q}$	$\rho ^{2}=0$		$\rho ^{2}=0.45$	
			B($\hat{Q}$) (*SD*)	Coverage percentage	B($\hat{Q}$) (*SD*)	Coverage percentage
100	Original	${\hat{{{\beta }}}}_{j}*$	.000 (.209)	.943	.002 (.080)	.945
	MCAR	${\bar{{\hat{\beta }}}}_{j,{\mathrm{PS}}}*$	.001 (.142)	.949	$-$.004 (.110)	.957
		${\bar{{\hat{\beta }}}}_{j,{\mathrm{SP}}}*$	.001 (.142)	.948	$-$.005 (.110)	.950
	MAR	${\bar{{\hat{\beta }}}}_{j,{\mathrm{PS}}}*$	$-$.001 (.119)	.945	.001 (.090)	.952
		${\bar{{\hat{\beta }}}}_{j,{\mathrm{SP}}}*$	$-$.001 (.119)	.945	.001 (.090)	.947
500	Original	${\hat{\beta }}_{j}*$	.000 (.047)	.947	.001 (.034)	.947
	MCAR	${\bar{{\hat{\beta }}}}_{j,{\mathrm{PS}}}*$	.000 (.058)	.948	.001 (.043)	.953
		${\bar{{\hat{\beta }}}}_{j,{\mathrm{SP}}}*$	.000 (.058)	.948	.001 (.043)	.949
	MAR	${\bar{{\hat{\beta }}}}_{j,{\mathrm{PS}}}*$	.000 (.049)	.950	.001 (.036)	.953
		${\bar{{\hat{\beta }}}}_{j,{\mathrm{SP}}}*$	.000 (.049)	.950	.001 (.036)	.950
100	Original	$$R^{2}* * $$	.042 (.028)		.016 (.076)	
	MCAR	$\hat{R}_{{\mathrm{PS}}}^{{2}}* *$	.065 (.048)		.007 (.099)	
		$\hat{R}_{{\mathrm{SP}}}^{{2}}* *$	.065 (.048)		.006 (.099)	
		${\mathfrak {R}}^{2}* *$	.087 (.052)		.034 (.096)	
		$\bar{R}^{2}* *$	.092 (.052)		.039 (.096)	
	MAR	$\hat{R}_{{\mathrm{PS}}}^{{2}}* *$	.050 (.035)		.006 (.084)	
		$\hat{R}_{{\mathrm{SP}}}^{{2}}* *$	.050 (.035)		.005 (.084)	
		${\mathfrak {R}}^{2}* *$	.059 (.037)		.025 (.082)	
		$\bar{R}^{2}* *$	.062 (.037)		.030 (.083)	
500	Original	$$R^{2}* * $$	.008 (.006)		.001 (.035)	
	MCAR	$\hat{R}_{{\mathrm{PS}}}^{{2}}* *$	.012 (.008)		$-$.001 (.041)	
		$\hat{R}_{{\mathrm{SP}}}^{{2}}* *$	.012 (.008)		$-$.001 (.041)	
		${\mathfrak {R}}^{2}* *$	.014 (.009)		.014 (.041)	
		$\bar{R}^{2}* *$	.016 (.009)		.020 (.041)	
	MAR	$\hat{R}_{{\mathrm{PS}}}^{{2}}* *$	.009 (.006)		$-$.003 (.037)	
		$\hat{R}_{{\mathrm{SP}}}^{{2}}* *$	.009 (.006)		$-$.003 (.037)	
		${\mathfrak {R}}^{2}* *$	.010 (.007)		.011 (.037)	
		$\bar{R}^{2}* *$	.010 (.007)		.016 (.037)	

For each outcome measure, results are shown for different sample sizes, missingness mechanisms, values of $\rho ^{2}$, and combination rules. Estimates of the original data are denoted ${\hat{\beta }}_{j}$ and $R^{2}.$

* Results in this row are aggregations across the remaining factors and predictors 2 to 4.

** Results in this row are aggregations across the remaining factors.

### Dependent Variables

The dependent variables in this study were 1) the biases in the sample estimates of parameters $\beta _{1}$ to $\beta _{4}$, 2) the number of times $\beta _{1}$ to $\beta _{4}$ were covered by the confidence intervals of their corresponding sample estimates, denoted coverage percentages, and 3) the bias in the sample estimate of $\rho ^{2}$. For comparison, the same outcome measures in the original data without missing values are reported as well.

## Results

When visually inspecting the results of the standardized regression coefficients, it turned out that with the exception of estimates and coverage percentages of $\beta _{1}$, results were very similar across standardized coefficients of the different predictors. Unlike the other $\beta _{j}$ estimates, estimates for $\beta _{1}$ had coverage percentages as low as 90% under $\rho ^{2}=0.455$ and a multivariate lognormal distribution of the predictors, for both the imputed data and the original data without missing values. Under a multivariate lognormal distribution, ${\mathbf {x}}_{1}$ was more heavily skewed to the right than the others (skewness of above 4 versus values around 1 for the other variables). Since Yuan and Chan (2011) point out that the coverages of the confidence intervals of standardized regression coefficients are influenced by the shape of the multivariate distribution of **X **as well, and the poor coverage of $\beta _{1}$ was found for both the imputed data and the original data without missing values, it was concluded that this was most probably not a problem of multiple imputation but of the delta method-based confidence interval itself. For this reason, it was decided to ignore this finding and focus only on the remaining three standardized regression coefficients.

Because of the size of the simulation design, a selection of factors to be displayed in a results table was made, based on effect sizes of ANOVAs.^1^ These effect sizes revealed that in general, the value of $\rho ^{2}$, sample size, missingness mechanism, and set of combination rules were the most influential factors. The results of the simulation study are given in Table 2 for all combinations of the different levels of these specific factors. Results for the estimated $\beta _{j}$s are aggregated across predictors, because it was found that differences in bias and coverage between different predictors were not substantial. The complete results are provided in supplemental material.

The table shows that in general, the differences between the estimates of ${\bar{{\hat{{\varvec{\upbeta }}}}}}_{\mathrm {PS}}$ and ${\bar{{\hat{{\varvec{\upbeta }}}}}}_{\mathrm {SP}}$ are negligibly small and often not even visible in the third decimal. In general, the bias in the estimated $\beta _{j}$s is close to 0. Additionally, for both methods ${\bar{{\hat{{\varvec{\upbeta }}}}}}_{\mathrm {PS}}$ and ${\bar{{\hat{{\varvec{\upbeta }}}}}}_{\mathrm {SP}}$, the coverage of the confidence intervals is close to the theoretical 95% in all situations.

Like the results for ${\bar{{\hat{{\varvec{\upbeta }}}}}}_{\mathrm {PS}}$ and ${\bar{{\hat{{\varvec{\upbeta }}}}}}_{\mathrm {SP}}$, the results for $\hat{R}_{\mathrm {PS}}^{{2}}$ and $\hat{R}_{\mathrm {SP}}^{{2}}$ are similar across all situations displayed in the table. However, there are clear differences between $\hat{R}_{\mathrm {PS}}^{{2}}$ and $\hat{R}_{\mathrm {SP}}^{{2}}$ on the one hand, and ${\mathfrak {R}}^{2}$ and $\bar{R^{2}}$ on the other hand. For the newly proposed estimates, the biases are substantially smaller than the biases of ${\mathfrak {R}}^{2}$ and $\bar{R^{2}}$ are.

## Discussion

In the current paper, two procedures for pooling standardized regression coefficients (${\bar{{\hat{\varvec{\upbeta }}}}}_{\mathrm {PS}}$ and ${\bar{{\hat{{\varvec{\upbeta }}}}}}_{\mathrm {SP}}$), and two new pooled estimates of $R^2$ ($\hat{R}_{\mathrm {PS}}^{{2}}$ and $\hat{R}_{\mathrm {SP}}^{{2}}$) in multiply imputed datasets, were proposed and investigated. Neither of the two procedures for standardized coefficients meets all the defined conditions for a statistic to be a standardized coefficient, as shown in Appendix II. However, simulation results showed that both methods produce very similar estimates and produce estimates that are close to the estimates of the original data and close to the corresponding population values. With the exception of $\beta _{1}$ under $\rho ^{2}=0.45$ and in skewed data, confidence intervals of both methods produced coverage percentages close to 95% for all other regression coefficients, and in all situations. Additionally, the two newly proposed pooled estimates of $R^{2}$, namely $\hat{R}_{\mathrm {PS}}^{{2}}$ and $\hat{R}_{\mathrm {SP}}^{{2}}$, outperformed the existing estimates ${\mathfrak {R}}^{2}$ and $\bar{R^{2}}$ in terms of bias. Both methods may thus be better alternatives than ${\mathfrak {R}}^{2}$ and $\bar{R^{2}}$.

Although the poor coverage of the confidence interval of $\beta _{1}$ in some situations may seem disappointing at first, it does not imply that the development of these combination rules for confidence intervals of standardized coefficients has been unsuccessful. Yuan and Chan (2011) already showed that even in complete data the coverage of the confidence intervals of the standardized regression coefficients depends on the distribution of the predictors. The fact that the coverage of $\beta _{1}$ under $\rho ^{2}=0.45$ and in skewed data was not any worse in the multiply imputed data than in the original data, suggests that this is a more of a problem of confidence intervals of standardized regression coefficients in general than of the proposed combination rules.

Additionally, it is worth mentioning that variable ${\mathbf {x}}_{1}$ did not have any missing data. Consequently, the estimates of $\beta _{1}$ and its confidence interval may have been less affected by imputed values and the combination rules than the estimates of the other standardized coefficients (although not entirely unaffected as imputed values on other variables will affect the estimation of the entire regression model, including $\beta _{1}$). The fact that coverage of $\beta _{1}$ was poor even though ${\mathbf {x}}_{1}$ did not contain any missing data while the coverages of other coefficients was satisfactory, makes an even stronger case for the hypothesis that the poor coverage of $\beta _{1}$ was caused by the distribution of ${\mathbf {x}}_{1}$ rather than by the proposed combination rules.

The poor results found for the coverage of $\beta _{1}$ do, however, raise the question how to obtain a valid confidence interval for standardized coefficients in multiply imputed data, when the predictors are highly skewed. In general, when analytic confidence intervals break down, bootstrap confidence intervals may provide a solution. Van Ginkel and Kiers (2011) proposed combination rules for bootstrap confidence intervals of principal component loadings. Their procedure may be generalizable to confidence intervals for standardized regression coefficients. However, whether these combination rules would give accurate coverage when applied to standardized coefficients is the topic of another study.

The remaining question is whether ${\bar{{\hat{{\varvec{\upbeta }}}}}}_{\mathrm {PS}}$ and $\hat{R}_{\mathrm {PS}}^{{2}}$ should be preferred, or ${\bar{{\hat{{\varvec{\upbeta }}}}}}_{\mathrm {SP}}$ and $\hat{R}_{\mathrm {SP}}^{{2}}$. This question is not easily answered. When looking at the conditions that have to be met for regression coefficients to be standardized coefficients, ${\bar{{\hat{{\varvec{\upbeta }}}}}}_{\mathrm {PS}}$ meets three of the four conditions, while ${\bar{{\hat{{\varvec{\upbeta }}}}}}_{\mathrm {SP}}$ meets only two. Using this as a criterion, it would follow that ${\bar{{\hat{{\varvec{\upbeta }}}}}}_{\mathrm {PS}}$ is the preferred method for pooling standardized regression coefficients and that the corresponding $\hat{R}_{\mathrm {PS}}^{{2}}\,$ is the preferred method for pooling $R^{2}$.

However, when looking at the specific conditions that ${\bar{{\hat{{\varvec{\upbeta }}}}}}_{\mathrm {SP}}$ does not satisfy, it may be wondered to what extent this is problematic. The first condition that ${\bar{{\hat{{\varvec{\upbeta }}}}}}_{\mathrm {SP}}$ does not satisfy is that a standardized regression coefficient can be computed using unbiased estimators of $b_{j}$, $\sigma _{x_{j}}^{2}$ and $\sigma _{y}^{2}$. This violation is mainly a problem from a theoretical point of view. As long as it does not cause substantial differences in simulation results between ${\bar{{\hat{{\varvec{\upbeta }}}}}}_{\mathrm {SP}}$ and ${\bar{{\hat{{\varvec{\upbeta }}}}}}_{\mathrm {PS}}$, the violation of this condition has little relevance.

The second condition that ${\bar{{\hat{{\varvec{\upbeta }}}}}}_{\mathrm {SP}}$ violates is that a *t*-test calculated for an unstandardized regression coefficient should be insensitive to standardization of the variables. On the one hand, this could be considered a disadvantage of ${\bar{{\hat{{\varvec{\upbeta }}}}}}_{\mathrm {SP}}$ compared to ${\bar{{\hat{{\varvec{\upbeta }}}}}}_{\mathrm {PS}}$. On the other hand, the fact that this condition is violated for ${\bar{{\hat{{\varvec{\upbeta }}}}}}_{\mathrm {SP}}$ actually illustrates an important property of the specific data transformation used for standardized regression coefficients. Although this transformation is linear, it is sample dependent because it depends on the sampling estimates $s_{{\mathbf {x}}_{j}}^{2}$ and $s_{{\mathbf {y}}}^{2}$. As these estimates get affected by the imputed values in multiply imputed data as well, it makes sense that the standardization is slightly different for each imputed dataset.

The different standardizations across imputed datasets may cause the pooled *t*-tests of the unstandardized regression coefficient to be different from the same pooled *t*-tests applied to the standardized variables. However, the fact that the specific standardization of variables is dependent on the sample, is a reason why a different *t*-test (Jones and Waller 2013) is used for standardized regression coefficients in the first place. When standardized regression coefficients are calculated for each imputed datasets in the same way as one would normally do in complete data, and Rubin’s rules are applied to the resulting coefficients and their correct standard errors next, this will automatically lead to the combination rules for point estimates and standard errors as defined by combination method ${\bar{{\hat{{\varvec{\upbeta }}}}}}_{\mathrm {SP}}$. Given that with the corrected standard error (Jones and Waller 2013) the sampling distribution of a standardized regression coefficient is (approximately) normal and Rubin’s combination rules have been defined under the assumption of a normal sampling distribution of the specific parameter, there seems to be no theoretical objection to using ${\bar{{\hat{{\varvec{\upbeta }}}}}}_{\mathrm {SP}}$ as a method for pooling standardized regression coefficients and their confidence intervals. The combination rules for the confidence intervals defined by method ${\bar{{\hat{{\varvec{\upbeta }}}}}}_{\mathrm {PS}}$, on the other hand, are not a direct application of Rubin’s rules to the standardized regression coefficients of each individual imputed dataset. Of course, this is only a problem from a theoretical point of view as the simulation results show that confidence intervals of methods ${\bar{{\hat{{\varvec{\upbeta }}}}}}_{\mathrm {PS}}$ and ${\bar{{\hat{{\varvec{\upbeta }}}}}}_{\mathrm {SP}}$ cover the population coefficients equally well.

Given that the theoretical objections to either method given so far are largely irrelevant when both methods perform equally well in terms of bias and coverage, the question is whether there is any reason left to prefer one method over the other. The answer to that question may lie in the only condition that the point estimates produced by ${\bar{{\hat{{\varvec{\upbeta }}}}}}_{\mathrm {PS}}$ do not satisfy. When simple regression is applied to a complete dataset, the estimates of $\beta _{1}$, $\beta _{1,y}$, and $\rho _{x_{1}y}$ are all equal. This is not the case for the estimates produced by ${\bar{{\hat{{\varvec{\upbeta }}}}}}_{\mathrm {PS}}$, while it is the case for ${\bar{{\hat{{\varvec{\upbeta }}}}}}_{\mathrm {SP}}$. For interpretational purposes, it may be important that researchers are not faced with inconsistencies among the estimates of $\beta _{1}$, $\beta _{1,y}$, and $\rho _{x_{1}y}$. Even when the differences are small, this could still cause some confusion on the part of the researcher. It could therefore be argued that ${\bar{{\hat{{\varvec{\upbeta }}}}}}_{\mathrm {SP}}$ and $\hat{R}_{\mathrm {SP}}^{{2}}$ are slightly preferred as pooled estimates for standardized coefficients and percentage of explained variance over ${\bar{{\hat{{\varvec{\upbeta }}}}}}_{\mathrm {PS}}$ and $\hat{R}_{\mathrm {PS}}^{{2}}$, respectively.

To conclude, this paper has proposed two sets of combination rules for standardized regression coefficients and their confidence intervals, and $R^{2}$ in multiply imputed datasets. Since the proposed methods all perform well in terms of bias and coverage, and the proposed methods for $R^{2}$ produce less bias than the earlier proposed measures of $R^{2}$ in various situations, one of these sets of combination rules may henceforth be used in future studies that use multiple regression in a multiply imputed dataset, with a slight preference for ${\bar{{\hat{{\varvec{\upbeta }}}}}}_{\mathrm {SP}}$ and $\hat{R}_{\mathrm {SP}}^{{2}}$.

### Electronic supplementary material

Below is the link to the electronic supplementary material.

Supplementary material 1 (docx 49 KB)

## Appendix I

Below proof will be given that in complete data the standardized coefficients meet the conditions stated in Table 1.

*Condition 1* For complete data, there is not much to prove about standardized coefficients satisfying Condition 1 as that condition already follows from the formula for standardized regression coefficients itself (Eq. 2).

*Condition 2* Suppose that we have a complete dataset with *N* cases, which consists of predictor variable matrix:

8 $$\begin{aligned} {\mathbf {X}}=\left[ {\begin{array}{llll} {1} &{} x_{11} &{} \ldots &{} x_{1k}\\ \vdots &{} \vdots &{} \vdots &{} \vdots \\ 1 &{} x_{Nk} &{} \ldots &{} x_{Nk}\\ \end{array} } \right] \mathbf {,} \end{aligned}$$

and outcome variable matrix:

$$\begin{aligned} {\mathbf {y}}=\left[ {\begin{array}{ll} {1} &{} y_{1}\\ \vdots &{} \vdots \\ 1 &{} y_{N}\\ \end{array} } \right] . \end{aligned}$$

${\mathbf {A}}_{{\mathbf {X}}}$ is a $(k + 1) \times (k + 1$) transformation matrix that transforms ${\mathbf {X}}$ into a predictor matrix with standardized variables. ${\mathbf {A}}_{{\mathbf {X}}}$ and ${{\mathbf {A}}^{\mathrm {'-1}}_{{\mathbf {X}}}}$ are defined as:

$$\begin{aligned} {\mathbf {A}}_{{\mathbf {X}}}=\left[ {\begin{array}{lllll} 1 &{} 0 &{} \ldots &{} \ldots &{} 0\\ -\frac{\bar{x}_{1}}{s_{{\mathbf {x}}_{1}}} &{} \frac{1}{s_{{\mathbf {x}}_{1}}} &{} 0 &{} \ldots &{} 0\\ \vdots &{} 0 &{} \ddots &{} \ddots &{} \vdots \\ \vdots &{} \vdots &{} \ddots &{} \ddots &{} 0\\ -\frac{\bar{x}_{k}}{s_{{\mathbf {x}}_{k}}} &{} 0 &{} \ldots &{} 0 &{} \frac{1}{s_{{\mathbf {x}}_{k}}}\\ \end{array} } \right] \quad \mathrm{and}\quad {{\mathbf {A}}^{\mathrm {'-1}}_{{\mathbf {X}}}}=\left[ {\begin{array}{lllll} 1 &{} \bar{x}_{1} &{} \ldots &{} \ldots &{} \bar{x}_{k}\\ 0 &{} s_{{\mathbf {x}}_{1}} &{} 0 &{} \ldots &{} 0\\ \vdots &{} 0 &{} \ddots &{} \ddots &{} \vdots \\ \vdots &{} \vdots &{} \ddots &{} \ddots &{} 0\\ 0 &{} \ldots &{} \ldots &{} 0 &{} s_{{\mathbf {x}}_{k}}\\ \end{array} } \right] , \end{aligned}$$

respectively.

Next, suppose that ${\mathbf {A}}_{{\mathbf {y}}}$ is a $2 \times 2$ transformation matrix that transforms ${\mathbf {y}}$ into a standardized outcome variable matrix. More specifically:

$$\begin{aligned} {\mathbf {A}}_{{\mathbf {y}}}=\left[ {\begin{array}{cc} 1 &{} 0\\ -\frac{\bar{y}}{s_{{\mathbf {y}}}} &{} \frac{1}{s_{{\mathbf {y}}}}\\ \end{array} } \right] ,\quad {{\mathbf {A}}^{\mathrm {'-1}}_{{\mathbf {y}}}}=\left[ {\begin{array}{cc} 1 &{} \bar{y}\\ 0 &{} s_{{\mathbf {y}}}\\ \end{array} } \right] . \end{aligned}$$

Standardized predictors and a standardized outcome variable may be obtained by means of:

$$\begin{aligned} {\mathbf {Z}}_{{\mathbf {X}}}= & {} {\mathbf {X}}{\mathbf {A}}_{{\mathbf {X}}}'=\left[ {\begin{array}{cccc} {1} &{}\frac{x_{11}-\bar{x}_{1}}{s_{{\mathbf {x}}_{1}}} &{} \ldots &{} \frac{x_{1k}-\bar{x}_{k}}{s_{{\mathbf {x}}_{k}}}\\ \vdots &{} \vdots &{} \vdots &{} \vdots \\ 1 &{} \frac{x_{N1}-\bar{x}_{1}}{s_{{\mathbf {x}}_{1}}} &{} \ldots &{} \frac{x_{Nk}-\bar{x}_{k}}{s_{{\mathbf {x}}_{k}}}\\ \end{array} } \right] ,\\ {\mathbf {Z}}_{{\mathbf {y}}}= & {} {\mathbf {y}}{\mathbf {A}}_{{\mathbf {y}}}'=\left[ {\begin{array}{cc} {1} &{} \frac{y_{1}-\bar{y}}{s_{{\mathbf {y}}}}\\ \vdots &{} \vdots \\ 1 &{} \frac{y_{N}-\bar{y}}{s_{{\mathbf {y}}}}\\ \end{array} } \right] . \end{aligned}$$

Using these standardized predictors, it can be shown that regression coefficients obtained from a regression using ${\mathbf {Z}}_{{\mathbf {X}}}$ and ${\mathbf {Z}}_{{\mathbf {y}}}$ as predictors and an outcome variable, respectively, will provide standardized regression coefficients, as defined in Eq. 2. First, the unstandardized regression coefficients are computed as:

$$\begin{aligned} {\hat{\mathbf {b}}}={({\mathbf {X}}'{\mathbf {X}})}^{-1} {\mathbf {X}}'{\mathbf {y}}. \end{aligned}$$

Equivalently, the standardized regression coefficients are computed as:

9 $$\begin{aligned} {\hat{{\varvec{\upbeta }}}}={({\mathbf {Z}}_{{\mathbf {X}}}' {\mathbf {Z}}_{{\mathbf {X}}})}^{-1} {\mathbf {Z}}_{{\mathbf {X}}}'{\mathbf {Z}}_{{\mathbf {y}}}. \end{aligned}$$

By substituting ${\mathbf {A}}_{{\mathbf {X}}}{\mathbf {X}}$ and ${\mathbf {y}}{\mathbf {A}}_{{\mathbf {y}}}$ for ${\mathbf {X}}$ and ${\mathbf {y}}$, respectively, it follows that Eqs. 2 and 9 are equivalent:

10 $$\begin{aligned} {\hat{{\varvec{\upbeta }}}}= & {} {({\mathbf {A}}_{{\mathbf {X}}} {\mathbf {X}}'{\mathbf {X}} {\mathbf {A}}_{{\mathbf {X}}}')}^{-1} {\mathbf {A}}_{{\mathbf {X}}}{\mathbf {X}}' {\mathbf {y}}{\mathbf {A}}_{{\mathbf {y}}}'\nonumber \\= & {} {{\mathbf {A}}_{{\mathbf {X}}}'}^{-1} {({\mathbf {X}}'{\mathbf {X}})}^{-1} {\mathbf {A}}_{{\mathbf {X}}}^{-1} {\mathbf {A}}_{{\mathbf {X}}}{\mathbf {X}}' {\mathbf {y}}{\mathbf {A}}_{{\mathbf {y}}}'\nonumber \\= & {} {{\mathbf {A}}_{{\mathbf {X}}}'}^{-1} {({\mathbf {X}}'{\mathbf {X}})}^{-1} {\mathbf {X}}'{\mathbf {y}}{\mathbf {A}}_{{\mathbf {y}}}'\nonumber \\= & {} {{\mathbf {A}}_{{\mathbf {X}}}'}^{-1} {\hat{\mathbf {b}}}{\mathbf {A}}_{{\mathbf {y}}}'\nonumber \\= & {} \left[ {\begin{array}{cc} 1 &{} 0\\ 0 &{} \frac{s_{{\mathbf {x}}_{1}}}{s_{{\mathbf {y}}}}{\hat{b}}_{1}\\ \vdots &{} \vdots \\ 0 &{} \frac{s_{{\mathbf {x}}_{k}}}{s_{{\mathbf {y}}}}{\hat{b}}_{k}\\ \end{array} } \right] \end{aligned}$$

(ignoring the first row and column, which are only meant for the intercepts in matrices ${\mathbf {X}}$ and ${\mathbf {y}}$).

*Condition 3* Before proving condition 3 for complete data, it is once again emphasized that the standard *t*-test for testing a regression coefficient is not suitable for standardized coefficients. Having said that, in complete data a linear transformation of the data does not affect the outcome of a *t*-test. Since standardizing the data is a linear transformation, the values of *t*-tests testing the resulting regression coefficients (which are now standardized coefficients) will not have been affected by this standardization. To show this, the formula for the *t*-test of a regression coefficient for unstandardized regression coefficients in complete data is given first. Suppose $s_{e}$ is the standard deviation of the error of the regression model, coefficient ${\hat{b}}_{j}$ is tested for significance the following *t*-statistic:

11 $$\begin{aligned} t=\frac{{\hat{b}}_{j}}{\frac{s_{e}}{s_{{\mathbf {x}}_{j}}\sqrt{(N-1)} }}=\frac{{\hat{b}}_{j}}{SE}\mathbf {,} \end{aligned}$$

with *n*– *k* – 1 degrees of freedom. Now suppose both the predictors and outcome variable are standardized, the (centered) values of the outcome variable are all divided by $s_{{\mathbf {y}}}$. Consequently, when a regression analysis is applied to these standardized variables, the standard deviation of the error in ${\mathbf {y}}$ becomes $s_{e,{\varvec{\upbeta }}}=\frac{s_{e}}{s_{{\mathbf {y}}}}$. Furthermore, the standardized coefficient of variable ${\mathbf {x}}_{j}$ is ${\hat{{{\beta }}}}_{j}=\frac{s_{{\mathbf {x}}_{j}}}{s_{{\mathbf {y}}}}{\hat{b}}_{j}$ (Eq. 2). Substituting these quantities into Eq. 11, and making use of the fact that $s_{{\mathbf {z}}_{j}}=1$, we get:

$$\begin{aligned} t= & {} \frac{\hat{\beta }_{j}}{\frac{s_{e,{{{\varvec{\upbeta }} }}}}{s_{{\mathbf {z}}_{j}}\sqrt{( N-1 )} }}{{=}}\frac{\frac{s_{\mathrm {\mathbf {x}}_{j}}}{s_{{\mathbf {y}}}}\hat{b}_{j}}{\frac{\frac{s_{e}}{s_{{\mathbf {y}}}}}{\sqrt{( N-1 )}}},\\= & {} \frac{\hat{b}_{j}}{\frac{s_{e}}{s_{{\mathbf {x}}_{j}}\sqrt{(N-1)} }}. \end{aligned}$$

*Condition 4* In simple linear regression performed on complete data, the standardized coefficient must equal the correlation between ${\mathbf {x}}_{1}$ and ${\mathbf {y}}$. An implication of this is that when ${\mathbf {y}}$ is regressed on ${\mathbf {x}}_{1}$, the standardized regression coefficient has the same value as the standardized regression coefficient that is obtained when ${\mathbf {x}}_{1}$ is regressed on ${\mathbf {y}}$, namely the correlation. If in complete data we regress variable ${\mathbf {x}}_1$ on variable ${\mathbf {y}}$, and we write $\, {\hat{{{\beta }}}}_{{1}}$ as a function of $r_{{\mathbf {x}}_{1}{\mathbf {y}}}$ we get:

$$\begin{aligned} \hat{\beta }_{{1}}= \frac{s_{{\mathbf {x}}_{1}}}{s_{{\mathbf {y}}}} {\hat{b}}_{1}=\frac{s_{{\mathbf {x}}_{1}}}{s_{{\mathbf {y}}}} \frac{s_{{\mathbf {y}}}}{s_{{\mathbf {x}}_{1}}} r_{{\mathbf {x}}_{1}{\mathbf {y}}} =r_{{\mathbf {x}}_{1}{\mathbf {y}}}. \end{aligned}$$

If we do the same for ${\bar{{\hat{{{\beta }}}}}}_{\mathrm {1,\mathbf {y}}}$ when we regress ${\mathbf {y}}$ on ${\mathbf {x}}$, we get:

$$\begin{aligned} {\hat{{\beta }}}_{\mathrm {1,\mathbf {y}}}= \frac{s_{{\mathbf {y}}}}{s_{{\mathbf {x}}_{1}}} \frac{s_{{\mathbf {x}}_{1}}}{s_{{\mathbf {y}}}} r_{{\mathbf {x}}_{1}{\mathbf {y}}} =r_{{\mathbf {x}}_{1}{\mathbf {y}}}. \end{aligned}$$

## Appendix II

Below it will be mathematically derived for both ${\bar{{\hat{{\varvec{\upbeta }}}}}}_{\mathrm {PS}}$ and ${\bar{{\hat{{\varvec{\upbeta }}}}}}_{\mathrm {SP}}$ whether they meet the conditions in Table 1.

*Condition 1* Assuming that the imputation model is Bayesianly proper (Schafer 1997, p. 105), it follows that ${\hat{b}}_{1,m}$, $s_{{\mathbf {x}}_{j},m}^{2}$ and $s_{{\mathbf {y}},m}^{2}$ in imputed dataset *m* are unbiased estimates of $b_{j}$, $\sigma _{{\mathbf {x}}_{j}}^{2}$ and $\sigma _{{\mathbf {y}}}^{2}$, respectively. Consequently, since $\mathrm {E}\left( \sum \nolimits _{m=1}^M {\hat{b}}_{j,m} \right) =\sum \nolimits _{m=1}^M {\mathrm {E}({\hat{b}}_{j,m})}$, it follows that

$$\begin{aligned} \frac{1}{M}\sum \limits _{m=1}^M {\mathrm {E}\left( {\hat{b}}_{j,m} \right) =} \frac{1}{M}\sum \limits _{m=1}^M {b_{j}=} b_{j}. \end{aligned}$$

The same can be shown for $\frac{1}{M}\sum \nolimits _{m=1}^M s_{{\mathbf {x}}_{j},m}^{2}$ and $\frac{1}{M}\sum \nolimits _{m=1}^M s_{{\mathbf {y}},m}^{2}$. Next, taking the square roots of the latter estimates will yield $\tilde{s}_{{\mathbf {x}}_{j}}$ and $\tilde{s}_{{\mathbf {y}}}$. Note that these estimates are not unbiased estimates of $\sigma _{{\mathbf {x}}_{j}}$ and $\sigma _{{\mathbf {y}}}$ as sample standard deviations are generally not unbiased estimators (Keeping 1962). However, this is also the case for $s_{{\mathbf {x}}_{j}}$ and $s_{{\mathbf {y}}}$ when there are no missing data. Thus, since $\tilde{s}_{{\mathbf {x}}_{1}}^{2}$ and $\tilde{s}_{{\mathbf {y}}}^{2}$ are unbiased estimates, the use of their square roots for the computation of ${\bar{{\hat{{\varvec{\upbeta }}}}}}_{\mathrm {PS}}$ will not introduce additional bias on top of the bias that a standardized regression coefficient has in itself. To summarize, since ${\bar{{\hat{b}}}}_{1}$, $\tilde{s}_{{\mathbf {x}}_{1}}^{2}$, and $\tilde{s}_{{\mathbf {y}}}^{2}$ are all unbiased estimates under Bayesianly proper imputations, ${\bar{{\hat{{\varvec{\upbeta }}}}}}_{\mathrm {PS}}$ satisfies condition 1.

Next, the question is whether ${\bar{{\hat{{\varvec{\upbeta }}}}}}_{\mathrm {SP}}$ satisfies condition 1 as well. Since all three statistics in the term $\frac{s_{{\mathbf {x}}_{j},m}}{s_{{\mathbf {y}},m}}{\hat{b}}_{j,m}$ behind the summation sign (Eq. 4) depend on *m*, no separate unbiased pooled estimates of $b_{j}$, $\sigma _{{\mathbf {x}}_{j}}^{2}$, and $\sigma _{{\mathbf {y}}}^{2}$ can be derived which, when inserted in Eq. 1, will provide the same pooled standardized regression coefficients as in Eq. 4. In short, ${\bar{{\hat{{\varvec{\upbeta }}}}}}_{\mathrm {SP}}$ does not satisfy condition 1.

*Condition 2* First, it is verified whether ${\bar{{\hat{{\varvec{\upbeta }}}}}}_{\mathrm {PS}}$ satisfies condition 2. Calculating $\tilde{s}_{{\mathbf {x}}_{j}}$ for all *j*, these estimates can be used to construct a transformation matrix for standardizing the set of predictors in imputed dataset *m*, denoted ${\mathbf {X}}_{m}$:

$$\begin{aligned} {\tilde{\mathbf {A}}}_{{\mathbf {X}}m}=\left[ {\begin{array}{lllll} 1 &{} 0 &{} \ldots &{} \ldots &{} 0\\ -\frac{\bar{x}_{1m}}{\tilde{s}_{{\mathbf {x}}_{1}}} &{} \frac{1}{\tilde{s}_{{\mathbf {x}}_{1}}} &{} 0 &{} \ldots &{} 0\\ \vdots &{} 0 &{} \ddots &{} \ddots &{} \vdots \\ \vdots &{} \vdots &{} \ddots &{} \ddots &{} 0\\ -\frac{\bar{x}_{km}}{\tilde{s}_{{\mathbf {x}}_{k}}} &{} 0 &{} \ldots &{} 0 &{} \frac{1}{\tilde{s}_{{\mathbf {x}}_{k}}}\\ \end{array} } \right] ,\quad {\tilde{{\mathbf {A}}}_{{\mathbf {X}}m}}^{\mathrm {'-1}}=\left[ {\begin{array}{lllll} 1 &{} \bar{x}_{1m} &{} \ldots &{} \ldots &{} \bar{x}_{km}\\ 0 &{} \tilde{s}_{{\mathbf {x}}_{1}} &{} 0 &{} \ldots &{} 0\\ \vdots &{} 0 &{} \ddots &{} \ddots &{} \vdots \\ \vdots &{} \vdots &{} \ddots &{} \ddots &{} 0\\ 0 &{} \ldots &{} \ldots &{} 0 &{} \tilde{s}_{{\mathbf {x}}_{k}}\\ \end{array} } \right] . \end{aligned}$$

Similarly, a transformation matrix for standardizing the outcome variable in imputed dataset *m*, denoted ${\mathbf {y}}_{m}$, can be constructed:

$$\begin{aligned} {\tilde{\mathbf {A}}}_{{\mathbf {y}}_{m}}=\left[ {\begin{array}{ll} 1 &{} 0\\ -\frac{\bar{y}_{m}}{\tilde{s}_{{\mathbf {y}}}} &{} \frac{1}{\tilde{s}_{{\mathbf {y}}}}\\ \end{array} } \right] \mathbf {,}\quad {\tilde{{\mathbf {A}}}_{{\mathbf {y}}_{m}}}^{\mathrm {'-1}}=\left[ {\begin{array}{cc} 1 &{} \bar{y}_{m}\\ 0 &{} \tilde{s}_{{\mathbf {y}}}\\ \end{array} } \right] . \end{aligned}$$

The standardized versions of ${\mathbf {X}}_{m}$ and ${\mathbf {y}}_{m}$ are computed as ${\tilde{\mathbf {Z}}}_{{\mathbf {X}}_{m}}={\mathbf {X}}{\tilde{\mathbf {A}}}_{{\mathbf {X}}_{m}}'$ and ${\tilde{\mathbf {Z}}}_{{\mathbf {y}}_{m}}={\mathbf {y}}{\tilde{\mathbf {A}}}_{{\mathbf {y}}_{m}}'$, respectively. Using the same steps as in Eq. 9 for complete data, it now follows that:

$$\begin{aligned} {\hat{{\varvec{\upbeta }}}}_{\mathrm {PS},m}=\left[ {\begin{array}{ll} 1 &{} 0\\ 0 &{} \frac{\tilde{s}_{{\mathbf {x}}_{1}}}{\tilde{s}_{{\mathbf {y}}}}{\hat{b}}_{1,m}\\ \vdots &{} \vdots \\ 0 &{} \frac{\tilde{s}_{{\mathbf {x}}_{k}}}{\tilde{s}_{{\mathbf {y}}}}{\hat{b}}_{k,m}\\ \end{array} } \right] . \end{aligned}$$

When averaging the ${\hat{{\varvec{\upbeta }}}}_{\mathrm {PS},m}\mathrm{'s}$ across the *M* imputed datasets, we get:

$$\begin{aligned} {\bar{{\hat{{\varvec{\upbeta }}}}}}_{\mathrm {PS}}=\frac{1}{M}\sum \limits _{m=1}^M {{\hat{{\varvec{\upbeta }}}}_{\mathrm {PS},m}=\frac{1}{M}\left[ {\begin{array}{cc} M &{} 0\\ 0 &{} \sum \limits _{m=1}^M {\frac{\tilde{s}_{{\mathbf {x}}_{1}}}{\tilde{s}_{{\mathbf {y}}}}{\hat{b}}_{1m}} \\ \vdots &{} \vdots \\ 0 &{} \sum \limits _{m=1}^M {\frac{\tilde{s}_{{\mathbf {x}}_{k}}}{\tilde{s}_{{\mathbf {y}}}}{\hat{b}}_{km}} \\ \end{array} } \right] } =\left[ {\begin{array}{cc} 1 &{} 0\\ 0 &{} \frac{\tilde{s}_{{\mathbf {x}}_{1}}}{\tilde{s}_{{\mathbf {y}}}}{\bar{{\hat{b}}}}_{1m}\\ \vdots &{} \vdots \\ 0 &{} \frac{\tilde{s}_{{\mathbf {x}}_{k}}}{\tilde{s}_{{\mathbf {y}}}}{\bar{{\hat{b}}}}_{km}\\ \end{array} } \right] , \end{aligned}$$

which is equivalent to Eq. 3, except for the first row and column, which are omitted in Eq. 3.

The idea behind this standardization is that within each imputed dataset the variable means of the specific imputed dataset *m* are used for the centering to ensure that the pooled intercept will be 0. At the same time, the normalization is done by dividing the same estimates of the standard deviations across imputed datasets to ensure that the resulting standardized regression coefficients will be the same as in Eq. 3.

This type of standardizing data is similar to standardization that is used in three-way models (Kroonenberg 2008, p. 127), where centering takes place at the level of fibers within a slice (in multiple imputation this corresponds with the computation the mean of a variable ${\mathbf {x}}_{j}$ within a specific imputed dataset *m*), and normalization takes place at the level of the complete slice (in multiple imputation, this corresponds with computing one standard deviation of a variable ${\mathbf {x}}_{j}$ across all imputed datasets). To summarize, when using a specific form of standardization and applying Rubin’s rules for point estimates (i.e., averaging), ${\bar{{\hat{{\varvec{\upbeta }}}}}}_{\mathrm {PS}}$ satisfies condition 2 as well.

As for ${\bar{{\hat{{\varvec{\upbeta }}}}}}_{\mathrm {SP}}$, the only difference with ${\bar{{\hat{{\varvec{\upbeta }}}}}}_{\mathrm {PS}}$ is that in the standardization both the centering and normalization take place at the level of imputed dataset *m*. The standardized regression coefficients in ${\bar{{\hat{{\varvec{\upbeta }}}}}}_{\mathrm {SP}}$ are the result of standardizing the predictors and outcome variable per imputed dataset *m*, applying a regression analysis to the standardized variables for each imputed dataset, and pooling the resulting regression coefficients using Rubin’s rules for point estimates (i.e., averaging). In short, using the general combination rules, ${\bar{{\hat{{\varvec{\upbeta }}}}}}_{\mathrm {SP}}$ satisfies condition 2 as well.

*Condition 3* When *t*-tests are calculated for ${\bar{{\hat{{\varvec{\upbeta }}}}}}_{\mathrm {PS}}$ in multiply imputed data using Rubin’s rules (1987, Chap. 3), the question is whether they will give the same *t*-tests as when the unstandardized coefficients in ${\bar{{\hat{\mathbf {b}}}}}$ are tested for significance using the same rules. When applying Rubin’s rules to an element ${\bar{{\hat{b}}}}_{j}$ in ${\bar{{\hat{\mathbf {b}}}}}$, we get:

$$\begin{aligned} \bar{Q}= & {} \frac{1}{M}\sum \limits _{m=1}^M {\hat{b}}_{j,m} ={\bar{{\hat{b}}}}_{j}\\ \bar{U}= & {} \frac{1}{M}\sum \limits _{m=1}^M \frac{s_{e,m}^{2}}{s_{{\mathbf {x}}_{j,m}}^{2}(N-1)}\\ B= & {} \frac{1}{M-1}\sum \limits _{m=1}^M {({\hat{b}}_{j,m}-{\bar{{\hat{b}}}}_{j})}^{2} =Var({\hat{b}}_{j})\\ T= & {} \frac{1}{M}\sum \limits _{m=1}^M \frac{s_{e,m}^{2}}{s_{{\mathbf {x}}_{j,m}}^{2}(N-1)} +\left( 1+M^{-1} \right) Var\left( {\hat{b}}_{j} \right) ={SE}^{2}\\ t= & {} \frac{{\bar{{\hat{b}}}}_{j}}{SE}. \end{aligned}$$

When we do the same for an element ${\bar{{\hat{\beta }}}}_{j\mathrm {,PS}}$ in ${\bar{{\hat{{\varvec{\upbeta }}}}}}_{\mathrm {PS}}$, we get:

$$\begin{aligned} \bar{Q}= & {} \frac{1}{M}\sum \limits _{m=1}^M {\frac{\tilde{s}_{{\mathbf {x}}_{j}}}{\tilde{s}_{{\mathbf {y}}}}{\hat{b}}_{j,m}} ={\frac{\tilde{s}_{{\mathbf {x}}_{j}}}{\tilde{s}_{{\mathbf {y}}}}{\bar{\hat{b}}}_{j}}\\ \bar{U}= & {} \frac{1}{M}\sum \limits _{m=1}^M \frac{\frac{s_{e,m}^{2}}{\tilde{s}_{{\mathbf {y}}}^{2}}}{\frac{s_{{\mathbf {x}}_{j},m}^{2}}{\tilde{s}_{{\mathbf {x}}_{j}}^{2}}(N-1)} =\frac{\tilde{s}_{{\mathbf {x}}_{j}}^{2}}{\tilde{s}_{{\mathbf {y}}}^{2}}\frac{1}{M}\sum \limits _{m=1}^M \frac{s_{e,m}^{2}}{s_{{\mathbf {x}}_{j,m}}^{2}(N-1)}\\ B= & {} \frac{1}{M-1}\sum \limits _{m=1}^M \left( {\frac{\tilde{s}_{{\mathbf {x}}_{j}}}{\tilde{s}_{{\mathbf {y}}}}{\hat{b}}_{j,m}}-\frac{\tilde{s}_{{\mathbf {x}}_{j}}}{\tilde{s}_{{\mathbf {y}}}}{\bar{{\hat{b}}}}_{j} \right) ^{2} =\frac{\tilde{s}_{{\mathbf {x}}_{j}}^{2}}{\tilde{s}_{{\mathbf {y}}}^{2}}Var\left( {\hat{b}}_{j} \right) ,\\ T= & {} \frac{\tilde{s}_{{\mathbf {x}}_{j}}^{2}}{\tilde{s}_{{\mathbf {y}}}^{2}}\frac{1}{M}\sum \limits _{m=1}^M \frac{s_{e,m}^{2}}{s_{{\mathbf {x}}_{j,m}}^{2}(N-1)} +\left( 1+M^{-1} \right) \frac{\tilde{s}_{{\mathbf {x}}_{j}}^{2}}{\tilde{s}_{{\mathbf {y}}}^{2}}Var\left( {\hat{b}}_{j} \right) ={\frac{\tilde{s}_{{\mathbf {x}}_{j}}^{2}}{\tilde{s}_{{\mathbf {y}}}^{2}}SE^{2}}\\ t= & {} \frac{{\frac{\tilde{s}_{{\mathbf {x}}_{j}}}{\tilde{s}_{{\mathbf {y}}}}{\bar{{\hat{b}}}}_{j}}}{\frac{\tilde{s}_{{\mathbf {x}}_{j}}}{\tilde{s}_{{\mathbf {y}}}}SE}=\frac{{\bar{{\hat{b}}}}_{j}}{SE}. \end{aligned}$$

In words, the term $\frac{\tilde{s}_{{\mathbf {x}}_{1}}^{2}}{\tilde{s}_{{\mathbf {y}}}^{2}}$ appears in both the estimate ${\bar{{\hat{{{\beta }}}}}}_{j\mathrm {,PS}}$ and its standard error. Since it does not depend on the imputation number *m*, it can be separated from the terms behind the summation sign, so that when ${\bar{{\hat{{{\beta }}}}}}_{j\mathrm {,PS}}$ is divided by its standard error, the terms $\frac{\tilde{s}_{{\mathbf {x}}_{j}}^{2}}{\tilde{s}_{{\mathbf {y}}}^{2}}$ in both the numerator and denominator cancel each other out. The resulting pooled *t*-test will then reduce to the *t*-test for unstandardized regression coefficients. The same cannot be done for ${\bar{{\hat{{\varvec{\upbeta }}}}}}_{\mathrm {SP}}$ because the term $\frac{s_{{\mathbf {x}}_{j},m}}{\tilde{s}_{{\mathbf {y}},m}}$ in Eq. 4 depends also on the imputation number. In summary, ${\bar{{\hat{{\varvec{\upbeta }}}}}}_{\mathrm {PS}}$ satisfies condition 3 and ${\bar{{\hat{{\varvec{\upbeta }}}}}}_{\mathrm {SP}}$ does not.

*Condition 4* To see whether this condition holds for ${\bar{{\hat{{\varvec{\upbeta }}}}}}_{\mathrm {PS}}$, we first write the pooled unstandardized coefficient ${\bar{{\hat{b}}}}_{1}$ as a function of the correlations of each of the *M* imputed datasets:

$$\begin{aligned} {\bar{{\hat{b}}}}_{1}=\frac{1}{M}\sum \limits _{m=1}^M {\frac{s_{{\mathbf {y}},m}}{s_{{\mathbf {x}}_{1},m}} r_{{\mathbf {x}}_{1}{\mathbf {y}},m}} . \end{aligned}$$

The standardized coefficient ${\bar{{\hat{{{\beta }}}}}}_{\mathrm {1,PS}}$ can be written as a function of $r_{{\mathbf {x}}_{1}{\mathbf {y}},m}$ as follows:

12 $$\begin{aligned} \bar{\hat{\beta }}_{\mathrm {1,PS}} =\frac{\tilde{s}_{{\mathbf {x}}_{1}}}{\tilde{s}_{{\mathbf {y}}}} \frac{1}{M}\sum \limits _{m=1}^M {\frac{s_{{\mathbf {y}},m}}{s_{{\mathbf {x}}_{1},m}} r_{{\mathbf {x}}_{1}{\mathbf {y}},m}}. \end{aligned}$$

If we do the same for the standardized regression coefficient of ${\mathbf {y}}$ on ${\mathbf {x}}_{1}$, denoted ${\bar{{\hat{{{\beta }}}}}}_{\mathrm {1,y,PS}}$, we get:

$$\begin{aligned} {\bar{{\hat{{{\beta }}}}}}_{\mathrm {1,y,PS}}=\frac{\tilde{s}_{{\mathbf {y}}}}{\tilde{s}_ {{\mathbf {x}}_{1}}} \frac{1}{M}\sum \limits _{m=1}^M {\frac{s_{{\mathbf {x}}_{1},m}}{s_{{\mathbf {y}},m}}r_{{\mathbf {x}}_{1}{\mathbf {y}},m}}, \end{aligned}$$

which is not equal to ${\bar{{\hat{{{\beta }}}}}}_{\mathrm {1,PS}}$ in Eq. 12. Thus, ${\bar{{\hat{{\varvec{\upbeta }}}}}}_{\mathrm {PS}}$ does not satisfy condition 4.

In contrast, if we write $\, {\bar{{\hat{{{\beta }}}}}}_{\mathrm {1,SP}}$ as a function of $r_{{\mathbf {x}}_{1}{\mathbf {y}},m}$, we get:

$$\begin{aligned} {\bar{{\hat{{{\beta }}}}}}_{\mathrm {1,SP}}=\frac{1}{M}\sum \limits _{m=1}^M \frac{s_{{\mathbf {x}}_{1},m}}{s_{{\mathbf {y}},m}} { {\hat{{{b }}}}}_{1,m}=\frac{1}{M}\sum \limits _{m=1}^M {\frac{s_{{\mathbf {x}}_{1},m}}{s_{{\mathbf {y}},m}} \frac{s_{{\mathbf {y}},m}}{s_{{\mathbf {x}}_{1},m}} r_{{\mathbf {x}}_{1}{\mathbf {y}},m}} =\frac{1}{M}\sum \limits _{m=1}^M r_{{\mathbf {x}}_{1}{\mathbf {y}},m} . \end{aligned}$$

If we do the same for ${\bar{{\hat{{{\beta }}}}}}_{\mathrm {1,\mathbf {y}}}$ , we get:

$$\begin{aligned} {\bar{\hat{{\beta }}}}_{\mathrm {1,y,SP}}=\frac{1}{M}\sum \limits _{m=1}^M {\frac{s_{{\mathbf {y}},m}}{s_{{\mathbf {x}}_{1},m}} \frac{s_{{\mathbf {x}}_{1},m}}{s_{{\mathbf {y}},m}} r_{{\mathbf {x}}_{1}{\mathbf {y}},m}} =\frac{1}{M}\sum \limits _{m=1}^M r_{{\mathbf {x}}_{1}{\mathbf {y}},m}. \end{aligned}$$

In other words, in simple regression, both ${\bar{{\hat{{{\beta }}}}}}_{\mathrm {1,SP}}$ and $\, {\bar{{\hat{{{\beta }}}}}}_{\mathrm {1,y,SP}}$ will give the same value, namely the average correlation across imputed datasets. Thus, ${\bar{{\hat{{\varvec{\upbeta }}}}}}_{\mathrm {SP}}$ meets condition 4 in the sense that regressing ${\mathbf {y}}$ on ${\mathbf {x}}_{1}$ and regressing ${\mathbf {x}}_{1}$ on ${\mathbf {y}}$ give the same pooled standardized coefficient.

## Footnotes

1 Without getting into too much detail, a specific factor was reported in the table of results if its effect or any of its interaction effect with other factors, on any of the outcome measures, was large according to Cohen (1988; partial $\eta ^{2} > 0.14$).
