# Supplementary material for: Standardized Regression Coefficients and Newly Proposed Estimators for R2 in Multiply Imputed Data
Source: Psychometrika. 2020 Mar 11;85(1):185–205. doi: 10.1007/s11336-020-09696-4 (PMC7186259; doi:10.1007/s11336-020-09696-4)
Supplement: Supplementary file 1 — Supplementary material 1 (docx 49 KB) [file 11336_2020_9696_MOESM1_ESM.docx]

Table 4: *Bias of standardized regression coefficient of predictor* $\mathbf{x}_{1}$ *(standard deviations between brackets). Entries must be multiplied by* 10^-3^.

| *ρ*² | *N* | Distr. | Miss.  Mech. | Comb.  Method | Percentage of missingness | | | |
| --- | --- | --- | --- | --- | --- | --- | --- | --- |
|  |  |  |  |  | 0% | 12.5% | 25% | 50% |
| 0 | 100 | Normal | MCAR | ${\bar{\hat{\beta}}}_{1,\mathrm{PS}}$ | -3 (116) | -1 (125) | -9 (140) | -1 (146) |
|  |  |  |  | ${\bar{\hat{\beta}}}_{1,\mathrm{SP}}$ |  | -1 (125) | -9 (140) | -1 (146) |
|  |  |  | MAR | ${\bar{\hat{\beta}}}_{1,\mathrm{PS}}$ |  | -3 (128) | -3 (134) | -1 (134) |
|  |  |  |  | ${\bar{\hat{\beta}}}_{1,\mathrm{SP}}$ |  | -3 (128) | -3 (133) | -1 (134) |
|  |  | Log  Normal | MCAR | ${\bar{\hat{\beta}}}_{1,\mathrm{PS}}$ | -3 (114) | -4 (121) | -2 (125) | -1 (131) |
|  |  |  |  | ${\bar{\hat{\beta}}}_{1,\mathrm{SP}}$ |  | -4 (121) | -2 (125) | -1 (131) |
|  |  |  | MAR | ${\bar{\hat{\beta}}}_{1,\mathrm{PS}}$ |  | -1 (122) | -2 (125) | -5 (120) |
|  |  |  |  | ${\bar{\hat{\beta}}}_{1,\mathrm{SP}}$ |  | -1 (122) | -2 (125) | -5 (119) |
|  | 500 | Normal | MCAR | ${\bar{\hat{\beta}}}_{1,\mathrm{PS}}$ | 1 (51) | 0 (55) | 1 (57) | -1 (57) |
|  |  |  |  | ${\bar{\hat{\beta}}}_{1,\mathrm{SP}}$ |  | 0 (55) | 1 (57) | -1 (57) |
|  |  |  | MAR | ${\bar{\hat{\beta}}}_{1,\mathrm{PS}}$ |  | 1 (54) | 0 (55) | 1 (55) |
|  |  |  |  | ${\bar{\hat{\beta}}}_{1,\mathrm{SP}}$ |  | 1 (54) | 0 (55) | 1 (55) |
|  |  | Log Normal | MCAR | ${\bar{\hat{\beta}}}_{1,\mathrm{PS}}$ | 1 (48) | 2 (53) | 0 (53) | 2 (53) |
|  |  |  |  | ${\bar{\hat{\beta}}}_{1,\mathrm{SP}}$ |  | 2 (53) | 0 (53) | 2 (53) |
|  |  |  | MAR | ${\bar{\hat{\beta}}}_{1,\mathrm{PS}}$ |  | 0 (55) | 1 (52) | 2 (51) |
|  |  |  |  | ${\bar{\hat{\beta}}}_{1,\mathrm{SP}}$ |  | 0 (55) | 1 (52) | 2 (51) |
| 0.45 | 100 | Normal | MCAR | ${\bar{\hat{\beta}}}_{1,\mathrm{PS}}$ | -3 (85) | -2 (98) | -7 (112) | -4 (114) |
|  |  |  |  | ${\bar{\hat{\beta}}}_{1,\mathrm{SP}}$ |  | -2 (98) | -7 (112) | -4 114) |
|  |  |  | MAR | ${\bar{\hat{\beta}}}_{1,\mathrm{PS}}$ |  | -2 (99) | -3 (104) | -3 (103) |
|  |  |  |  | ${\bar{\hat{\beta}}}_{1,\mathrm{SP}}$ |  | -2 (99) | -3 (104) | -3 (103) |
|  |  | Log Normal | MCAR | ${\bar{\hat{\beta}}}_{1,\mathrm{PS}}$ | -10 (98) | -13 (108) | -17 (117) | -18 (123) |
|  |  |  |  | ${\bar{\hat{\beta}}}_{1,\mathrm{SP}}$ |  | -13 (108) | -17 (117) | -18 (123) |
|  |  |  | MAR | ${\bar{\hat{\beta}}}_{1,\mathrm{PS}}$ |  | -43 (118) | -43 (121) | -44 (119) |
|  |  |  |  | ${\bar{\hat{\beta}}}_{1,\mathrm{SP}}$ |  | -43 (118) | -44 (121) | -45 (119) |
|  | 500 | Normal | MCAR | ${\bar{\hat{\beta}}}_{1,\mathrm{PS}}$ | 1 (37) | 0 (42) | 0 (45) | 0 (44) |
|  |  |  |  | ${\bar{\hat{\beta}}}_{1,\mathrm{SP}}$ |  | 0 (42) | 0 (45) | 0 (44) |
|  |  |  | MAR | ${\bar{\hat{\beta}}}_{1,\mathrm{PS}}$ |  | 1 (41) | 0 (42) | 1 (41) |
|  |  |  |  | ${\bar{\hat{\beta}}}_{1,\mathrm{SP}}$ |  | 1 (41) | 0 (42) | 1 (41) |
|  |  | Log Normal | MCAR | ${\bar{\hat{\beta}}}_{1,\mathrm{PS}}$ | 0 (45) | -3 (49) | -5 (51) | -4 (50) |
|  |  |  |  | ${\bar{\hat{\beta}}}_{1,\mathrm{SP}}$ |  | -3 (49) | -5 (51) | -4 (50) |
|  |  |  | MAR | ${\bar{\hat{\beta}}}_{1,\mathrm{PS}}$ |  | -15 (49) | -15 (49) | -13 (50) |
|  |  |  |  | ${\bar{\hat{\beta}}}_{1,\mathrm{SP}}$ |  | -15 (49) | -15 (49) | -13 (50) |

Table 5: *Results coverage percentages of standardized regression coefficient of predictor* $\mathbf{x}_{1}$.

| *ρ*² | *N* | Distribution | Missingness  Mechanism | Combination Method | Percentage of missingness | | | |
| --- | --- | --- | --- | --- | --- | --- | --- | --- |
|  |  |  |  |  | 0% | 12.5% | 25% | 50% |
| 0 | 100 | Normal | MCAR | ${\bar{\hat{\beta}}}_{1,\mathrm{PS}}$ | .944 | .947 | .953 | .949 |
|  |  |  |  | ${\bar{\hat{\beta}}}_{1,\mathrm{SP}}$ |  | .945 | .945 | .945 |
|  |  |  | MAR | ${\bar{\hat{\beta}}}_{1,\mathrm{PS}}$ |  | .949 | .940 | .938 |
|  |  |  |  | ${\bar{\hat{\beta}}}_{1,\mathrm{SP}}$ |  | .948 | .938 | .936 |
|  |  | Log Normal | MCAR | ${\bar{\hat{\beta}}}_{1,\mathrm{PS}}$ | .931 | .949 | .950 | .929 |
|  |  |  |  | ${\bar{\hat{\beta}}}_{1,\mathrm{SP}}$ |  | .949 | .948 | .928 |
|  |  |  | MAR | ${\bar{\hat{\beta}}}_{1,\mathrm{PS}}$ |  | .953 | .953 | .960 |
|  |  |  |  | ${\bar{\hat{\beta}}}_{1,\mathrm{SP}}$ |  | .952 | .952 | .960 |
|  | 500 | Normal | MCAR | ${\bar{\hat{\beta}}}_{1,\mathrm{PS}}$ | .950 | .952 | .952 | .952 |
|  |  |  |  | ${\bar{\hat{\beta}}}_{1,\mathrm{SP}}$ |  | .952 | .952 | .952 |
|  |  |  | MAR | ${\bar{\hat{\beta}}}_{1,\mathrm{PS}}$ |  | .952 | .946 | .947 |
|  |  |  |  | ${\bar{\hat{\beta}}}_{1,\mathrm{SP}}$ |  | .952 | .946 | .946 |
|  |  | Log Normal | MCAR | ${\bar{\hat{\beta}}}_{1,\mathrm{PS}}$ | .946 | .948 | .954 | .957 |
|  |  |  |  | ${\bar{\hat{\beta}}}_{1,\mathrm{SP}}$ |  | .947 | .954 | .957 |
|  |  |  | MAR | ${\bar{\hat{\beta}}}_{1,\mathrm{PS}}$ |  | .945 | .963 | .962 |
|  |  |  |  | ${\bar{\hat{\beta}}}_{1,\mathrm{SP}}$ |  | .944 | .963 | .962 |
| 0.45 | 100 | Normal | MCAR | ${\bar{\hat{\beta}}}_{1,\mathrm{PS}}$ | .946 | .951 | .957 | .963 |
|  |  |  |  | ${\bar{\hat{\beta}}}_{1,\mathrm{SP}}$ |  | .948 | .955 | .957 |
|  |  |  | MAR | ${\bar{\hat{\beta}}}_{1,\mathrm{PS}}$ |  | .956 | .950 | .958 |
|  |  |  |  | ${\bar{\hat{\beta}}}_{1,\mathrm{SP}}$ |  | .953 | .947 | .951 |
|  |  | Log Normal | MCAR | ${\bar{\hat{\beta}}}_{1,\mathrm{PS}}$ | .890 | .915 | .930 | .918 |
|  |  |  |  | ${\bar{\hat{\beta}}}_{1,\mathrm{SP}}$ |  | .913 | .924 | .913 |
|  |  |  | MAR | ${\bar{\hat{\beta}}}_{1,\mathrm{PS}}$ |  | .939 | .936 | .935 |
|  |  |  |  | ${\bar{\hat{\beta}}}_{1,\mathrm{SP}}$ |  | .937 | .932 | .930 |
|  | 500 | Normal | MCAR | ${\bar{\hat{\beta}}}_{1,\mathrm{PS}}$ | .947 | .946 | .946 | .956 |
|  |  |  |  | ${\bar{\hat{\beta}}}_{1,\mathrm{SP}}$ |  | .944 | .942 | .952 |
|  |  |  | MAR | ${\bar{\hat{\beta}}}_{1,\mathrm{PS}}$ |  | .956 | .944 | .946 |
|  |  |  |  | ${\bar{\hat{\beta}}}_{1,\mathrm{SP}}$ |  | .952 | .943 | .941 |
|  |  | Log Normal | MCAR | ${\bar{\hat{\beta}}}_{1,\mathrm{PS}}$ | .891 | .899 | .900 | .904 |
|  |  |  |  | ${\bar{\hat{\beta}}}_{1,\mathrm{SP}}$ |  | .897 | .897 | .903 |
|  |  |  | MAR | ${\bar{\hat{\beta}}}_{1,\mathrm{PS}}$ |  | .923 | .918 | .920 |
|  |  |  |  | ${\bar{\hat{\beta}}}_{1,\mathrm{SP}}$ |  | .922 | .916 | .916 |

Table 6: *Bias of standardized regression coefficient of predictor* $\mathbf{x}_{2}$ *(standard deviations between brackets). Entries must be multiplied by* 10^-3^.

| *ρ*² | *N* | Distr. | Miss.  Mech. | Comb.  Method | Percentage of missingness | | | |
| --- | --- | --- | --- | --- | --- | --- | --- | --- |
|  |  |  |  |  | 0% | 12.5% | 25% | 50% |
| 0 | 100 | Normal | MCAR | ${\bar{\hat{\beta}}}_{2,\mathrm{PS}}$ | -1 (105) | -5 (127) | 3 (156) | 2 (154) |
|  |  |  |  | ${\bar{\hat{\beta}}}_{2,\mathrm{SP}}$ |  | -5 (127) | 3 (155) | 2 (153) |
|  |  |  | MAR | ${\bar{\hat{\beta}}}_{2,\mathrm{PS}}$ |  | -3 (120) | -1 (125) | -2 (124) |
|  |  |  |  | ${\bar{\hat{\beta}}}_{2,\mathrm{SP}}$ |  | -3 (120) | -1 (125) | -2 (124) |
|  |  | Log  Normal | MCAR | ${\bar{\hat{\beta}}}_{2,\mathrm{PS}}$ | 1 (104) | 0 (119) | -3 (135) | -7 (132) |
|  |  |  |  | ${\bar{\hat{\beta}}}_{2,\mathrm{SP}}$ |  | 0 (119) | -3 (135) | -6 (132) |
|  |  |  | MAR | ${\bar{\hat{\beta}}}_{2,\mathrm{PS}}$ |  | 0 (106) | 1 (107) | 0 (106) |
|  |  |  |  | ${\bar{\hat{\beta}}}_{2,\mathrm{SP}}$ |  | 0 (106) | 1 (106) | 0 (106) |
|  | 500 | Normal | MCAR | ${\bar{\hat{\beta}}}_{2,\mathrm{PS}}$ | -2 (46) | -1 (55) | -2 (59) | -2 (60) |
|  |  |  |  | ${\bar{\hat{\beta}}}_{2,\mathrm{SP}}$ |  | -1 (55) | -2 (59) | -2 (60) |
|  |  |  | MAR | ${\bar{\hat{\beta}}}_{2,\mathrm{PS}}$ |  | -2 (49) | -2 (49) | -2 (50) |
|  |  |  |  | ${\bar{\hat{\beta}}}_{2,\mathrm{SP}}$ |  | -2 (49) | -2 (49) | -2 (50) |
|  |  | Log Normal | MCAR | ${\bar{\hat{\beta}}}_{2,\mathrm{PS}}$ | 0 (46) | 0 (54) | 2 (55) | 3 (56) |
|  |  |  |  | ${\bar{\hat{\beta}}}_{2,\mathrm{SP}}$ |  | 0 (54) | 2 (55) | 3 (56) |
|  |  |  | MAR | ${\bar{\hat{\beta}}}_{2,\mathrm{PS}}$ |  | 0 (46) | 1 (46) | 0 (46) |
|  |  |  |  | ${\bar{\hat{\beta}}}_{2,\mathrm{SP}}$ |  | 0 (46) | 1 (46) | 0 (46) |
| 0.45 | 100 | Normal | MCAR | ${\bar{\hat{\beta}}}_{2,\mathrm{PS}}$ | 0 (77) | -2 (96) | 0 (116) | 0 (119) |
|  |  |  |  | ${\bar{\hat{\beta}}}_{2,\mathrm{SP}}$ |  | -3 (96) | 0 (116) | 0 (118) |
|  |  |  | MAR | ${\bar{\hat{\beta}}}_{2,\mathrm{PS}}$ |  | -2 (90) | -1 (93) | -2 (92) |
|  |  |  |  | ${\bar{\hat{\beta}}}_{2,\mathrm{SP}}$ |  | -2 (90) | -1 (93) | -2 (92) |
|  |  | Log Normal | MCAR | ${\bar{\hat{\beta}}}_{2,\mathrm{PS}}$ | 2 (80) | -2 (95) | -12 (116) | -15 (116) |
|  |  |  |  | ${\bar{\hat{\beta}}}_{2,\mathrm{SP}}$ |  | -2 (95) | -12 (115) | -16 (116) |
|  |  |  | MAR | ${\bar{\hat{\beta}}}_{2,\mathrm{PS}}$ |  | 1 (85) | 2 (85) | 1 (84) |
|  |  |  |  | ${\bar{\hat{\beta}}}_{2,\mathrm{SP}}$ |  | 1 (85) | 2 (85) | 1 (84) |
|  | 500 | Normal | MCAR | ${\bar{\hat{\beta}}}_{2,\mathrm{PS}}$ | -2 (34) | -2 (41) | -1 (46) | -1 (45) |
|  |  |  |  | ${\bar{\hat{\beta}}}_{2,\mathrm{SP}}$ |  | -2 (41) | -1 (46) | -1 (45) |
|  |  |  | MAR | ${\bar{\hat{\beta}}}_{2,\mathrm{PS}}$ |  | -2 (36) | -2 (36) | -1 (37) |
|  |  |  |  | ${\bar{\hat{\beta}}}_{2,\mathrm{SP}}$ |  | -2 (36) | -2 (36) | -1 (37) |
|  |  | Log Normal | MCAR | ${\bar{\hat{\beta}}}_{2,\mathrm{PS}}$ | 1 (35) | -1 (43) | -1 (44) | 1 (46) |
|  |  |  |  | ${\bar{\hat{\beta}}}_{2,\mathrm{SP}}$ |  | -1 (43) | -1 (44) | 1 (46) |
|  |  |  | MAR | ${\bar{\hat{\beta}}}_{2,\mathrm{PS}}$ |  | 1 (36) | 1 (36) | 1 (36) |
|  |  |  |  | ${\bar{\hat{\beta}}}_{2,\mathrm{SP}}$ |  | 1 (36) | 1 (36) | 1 (36) |

Table 7: *Results coverage percentages of standardized regression coefficient of predictor* $\mathbf{x}_{2}$.

| *ρ*² | *N* | Distribution | Missingness  Mechanism | Combination Method | Percentage of missingness | | | |
| --- | --- | --- | --- | --- | --- | --- | --- | --- |
|  |  |  |  |  | 0% | 12.5% | 25% | 50% |
| 0 | 100 | Normal | MCAR | ${\bar{\hat{\beta}}}_{2,\mathrm{PS}}$ | .952 | .946 | .947 | .947 |
|  |  |  |  | ${\bar{\hat{\beta}}}_{2,\mathrm{SP}}$ |  | .946 | .945 | .946 |
|  |  |  | MAR | ${\bar{\hat{\beta}}}_{2,\mathrm{PS}}$ |  | .952 | .946 | .947 |
|  |  |  |  | ${\bar{\hat{\beta}}}_{2,\mathrm{SP}}$ |  | .952 | .946 | .948 |
|  |  | Log Normal | MCAR | ${\bar{\hat{\beta}}}_{2,\mathrm{PS}}$ | .946 | .948 | .947 | .965 |
|  |  |  |  | ${\bar{\hat{\beta}}}_{2,\mathrm{SP}}$ |  | .949 | .945 | .965 |
|  |  |  | MAR | ${\bar{\hat{\beta}}}_{2,\mathrm{PS}}$ |  | .960 | .952 | .956 |
|  |  |  |  | ${\bar{\hat{\beta}}}_{2,\mathrm{SP}}$ |  | .959 | .953 | .956 |
|  | 500 | Normal | MCAR | ${\bar{\hat{\beta}}}_{2,\mathrm{PS}}$ | .942 | .950 | .948 | .941 |
|  |  |  |  | ${\bar{\hat{\beta}}}_{2,\mathrm{SP}}$ |  | .950 | .948 | .942 |
|  |  |  | MAR | ${\bar{\hat{\beta}}}_{2,\mathrm{PS}}$ |  | .946 | .941 | .949 |
|  |  |  |  | ${\bar{\hat{\beta}}}_{2,\mathrm{SP}}$ |  | .946 | .940 | .949 |
|  |  | Log Normal | MCAR | ${\bar{\hat{\beta}}}_{2,\mathrm{PS}}$ | .945 | .955 | .958 | .946 |
|  |  |  |  | ${\bar{\hat{\beta}}}_{2,\mathrm{SP}}$ |  | .954 | .957 | .948 |
|  |  |  | MAR | ${\bar{\hat{\beta}}}_{2,\mathrm{PS}}$ |  | .945 | .947 | .946 |
|  |  |  |  | ${\bar{\hat{\beta}}}_{2,\mathrm{SP}}$ |  | .945 | .947 | .946 |
| 0.45 | 100 | Normal | MCAR | ${\bar{\hat{\beta}}}_{2,\mathrm{PS}}$ | .952 | .966 | .957 | .952 |
|  |  |  |  | ${\bar{\hat{\beta}}}_{2,\mathrm{SP}}$ |  | .960 | .952 | .947 |
|  |  |  | MAR | ${\bar{\hat{\beta}}}_{2,\mathrm{PS}}$ |  | .950 | .951 | .956 |
|  |  |  |  | ${\bar{\hat{\beta}}}_{2,\mathrm{SP}}$ |  | .951 | .948 | .954 |
|  |  | Log Normal | MCAR | ${\bar{\hat{\beta}}}_{2,\mathrm{PS}}$ | .940 | .952 | .952 | .956 |
|  |  |  |  | ${\bar{\hat{\beta}}}_{2,\mathrm{SP}}$ |  | .948 | .953 | .949 |
|  |  |  | MAR | ${\bar{\hat{\beta}}}_{2,\mathrm{PS}}$ |  | .939 | .945 | .949 |
|  |  |  |  | ${\bar{\hat{\beta}}}_{2,\mathrm{SP}}$ |  | .936 | .941 | .946 |
|  | 500 | Normal | MCAR | ${\bar{\hat{\beta}}}_{2,\mathrm{PS}}$ | .949 | .952 | .952 | .942 |
|  |  |  |  | ${\bar{\hat{\beta}}}_{2,\mathrm{SP}}$ |  | .952 | .949 | .938 |
|  |  |  | MAR | ${\bar{\hat{\beta}}}_{2,\mathrm{PS}}$ |  | .956 | .950 | .954 |
|  |  |  |  | ${\bar{\hat{\beta}}}_{2,\mathrm{SP}}$ |  | .949 | .949 | .953 |
|  |  | Log Normal | MCAR | ${\bar{\hat{\beta}}}_{2,\mathrm{PS}}$ | .933 | .952 | .947 | .943 |
|  |  |  |  | ${\bar{\hat{\beta}}}_{2,\mathrm{SP}}$ |  | .945 | .951 | .938 |
|  |  |  | MAR | ${\bar{\hat{\beta}}}_{2,\mathrm{PS}}$ |  | .943 | .948 | .942 |
|  |  |  |  | ${\bar{\hat{\beta}}}_{2,\mathrm{SP}}$ |  | .943 | .945 | .941 |

Table 8: *Bias of standardized regression coefficient of predictor* $\mathbf{x}_{3}$ *(standard deviations between brackets). Entries must be multiplied by* 10^-3^.

| *ρ*² | *N* | Distr. | Miss.  Mech. | Comb.  Method | Percentage of missingness | | | |
| --- | --- | --- | --- | --- | --- | --- | --- | --- |
|  |  |  |  |  | 0% | 12.5% | 25% | 50% |
| 0 | 100 | Normal | MCAR | ${\bar{\hat{\beta}}}_{3,\mathrm{PS}}$ | 1 (108) | 4 (129) | 2 (151) | 2 (157) |
|  |  |  |  | ${\bar{\hat{\beta}}}_{3,\mathrm{SP}}$ |  | 4 (129) | 2 (151) | 2 (157) |
|  |  |  | MAR | ${\bar{\hat{\beta}}}_{3,\mathrm{PS}}$ |  | -2 (123) | 0 (126) | -1 (128) |
|  |  |  |  | ${\bar{\hat{\beta}}}_{3,\mathrm{SP}}$ |  | -2 (123) | 0 (126) | -1 (127) |
|  |  | Log  Normal | MCAR | ${\bar{\hat{\beta}}}_{3,\mathrm{PS}}$ | 1 (108) | 3 (123) | 6 (137) | 1 (141) |
|  |  |  |  | ${\bar{\hat{\beta}}}_{3,\mathrm{SP}}$ |  | 3 (123) | 6 (137) | 1 (141) |
|  |  |  | MAR | ${\bar{\hat{\beta}}}_{3,\mathrm{PS}}$ |  | 2 (110) | 1 (112) | 0 (112) |
|  |  |  |  | ${\bar{\hat{\beta}}}_{3,\mathrm{SP}}$ |  | 2 (110) | 1 (112) | 0 (112) |
|  | 500 | Normal | MCAR | ${\bar{\hat{\beta}}}_{3,\mathrm{PS}}$ | 1 (45) | 0 (55) | 1 (57) | -1 (57) |
|  |  |  |  | ${\bar{\hat{\beta}}}_{3,\mathrm{SP}}$ |  | 0 (55) | 1 (57) | -1 (57) |
|  |  |  | MAR | ${\bar{\hat{\beta}}}_{3,\mathrm{PS}}$ |  | 1 (48) | 1 (47) | 0 (48) |
|  |  |  |  | ${\bar{\hat{\beta}}}_{3,\mathrm{SP}}$ |  | 1 (48) | 1 (47) | 0 (48) |
|  |  | Log Normal | MCAR | ${\bar{\hat{\beta}}}_{3,\mathrm{PS}}$ | 2 (45) | 0 (53) | 0 (58) | 0 (56) |
|  |  |  |  | ${\bar{\hat{\beta}}}_{3,\mathrm{SP}}$ |  | 0 (53) | 0 (58) | 0 (56) |
|  |  |  | MAR | ${\bar{\hat{\beta}}}_{3,\mathrm{PS}}$ |  | 2 (46) | 2 (46) | 1 (46) |
|  |  |  |  | ${\bar{\hat{\beta}}}_{3,\mathrm{SP}}$ |  | 2 (46) | 2 (46) | 1 (46) |
| 0.45 | 100 | Normal | MCAR | ${\bar{\hat{\beta}}}_{3,\mathrm{PS}}$ | 1 (76) | 1 (90) | 0 (108) | 0 (111) |
|  |  |  |  | ${\bar{\hat{\beta}}}_{3,\mathrm{SP}}$ |  | 1 (90) | 0 (107) | -1 (112) |
|  |  |  | MAR | ${\bar{\hat{\beta}}}_{3,\mathrm{PS}}$ |  | -1 (87) | -3 (90) | -1 (90) |
|  |  |  |  | ${\bar{\hat{\beta}}}_{3,\mathrm{SP}}$ |  | -1 (87) | 3 (90) | -1 (90) |
|  |  | Log Normal | MCAR | ${\bar{\hat{\beta}}}_{3,\mathrm{PS}}$ | 3 (80) | -3 (93) | -9 (111) | -13 (113) |
|  |  |  |  | ${\bar{\hat{\beta}}}_{3,\mathrm{SP}}$ |  | -4 (93) | -10 (111) | -13 (113) |
|  |  |  | MAR | ${\bar{\hat{\beta}}}_{3,\mathrm{PS}}$ |  | 4 (83) | 4 (84) | 4 (83) |
|  |  |  |  | ${\bar{\hat{\beta}}}_{3,\mathrm{SP}}$ |  | 4 (83) | 4 (84) | 4 (83) |
|  | 500 | Normal | MCAR | ${\bar{\hat{\beta}}}_{3,\mathrm{PS}}$ | 1 (31) | 1 (37) | 0 (39) | 0 (38) |
|  |  |  |  | ${\bar{\hat{\beta}}}_{3,\mathrm{SP}}$ |  | 1 (37) | 0 (39) | 0 (38) |
|  |  |  | MAR | ${\bar{\hat{\beta}}}_{3,\mathrm{PS}}$ |  | 1 (33) | 1 (33) | 1 (33) |
|  |  |  |  | ${\bar{\hat{\beta}}}_{3,\mathrm{SP}}$ |  | 1 (33) | 1 (33) | 1 (33) |
|  |  | Log Normal | MCAR | ${\bar{\hat{\beta}}}_{3,\mathrm{PS}}$ | 3 (32) | 1 (38) | 1 (42) | 0 (40) |
|  |  |  |  | ${\bar{\hat{\beta}}}_{3,\mathrm{SP}}$ |  | 1 (38) | 1 (42) | 0 (40) |
|  |  |  | MAR | ${\bar{\hat{\beta}}}_{3,\mathrm{PS}}$ |  | 4 (33) | 4 (33) | 4 (33) |
|  |  |  |  | ${\bar{\hat{\beta}}}_{3,\mathrm{SP}}$ |  | 4 (33) | 4 (33) | 4 (33) |

Table 9: *Results coverage percentages of standardized regression coefficient of predictor* $\mathbf{x}_{1}$.

| *ρ*² | *N* | Distribution | Missingness  Mechanism | Combination Method | Percentage of missingness | | | |
| --- | --- | --- | --- | --- | --- | --- | --- | --- |
|  |  |  |  |  | 0% | 12.5% | 25% | 50% |
| 0 | 100 | Normal | MCAR | ${\bar{\hat{\beta}}}_{3,\mathrm{PS}}$ | .936 | .941 | .957 | .945 |
|  |  |  |  | ${\bar{\hat{\beta}}}_{3,\mathrm{SP}}$ |  | .940 | .953 | .940 |
|  |  |  | MAR | ${\bar{\hat{\beta}}}_{3,\mathrm{PS}}$ |  | .936 | .937 | .934 |
|  |  |  |  | ${\bar{\hat{\beta}}}_{3,\mathrm{SP}}$ |  | .936 | .936 | .930 |
|  |  | Log Normal | MCAR | ${\bar{\hat{\beta}}}_{3,\mathrm{PS}}$ | .931 | .944 | .952 | .958 |
|  |  |  |  | ${\bar{\hat{\beta}}}_{3,\mathrm{SP}}$ |  | .943 | .952 | .956 |
|  |  |  | MAR | ${\bar{\hat{\beta}}}_{3,\mathrm{PS}}$ |  | .935 | .930 | .933 |
|  |  |  |  | ${\bar{\hat{\beta}}}_{3,\mathrm{SP}}$ |  | .936 | .931 | .933 |
|  | 500 | Normal | MCAR | ${\bar{\hat{\beta}}}_{3,\mathrm{PS}}$ | .947 | .945 | .957 | .959 |
|  |  |  |  | ${\bar{\hat{\beta}}}_{3,\mathrm{SP}}$ |  | .946 | .957 | .958 |
|  |  |  | MAR | ${\bar{\hat{\beta}}}_{3,\mathrm{PS}}$ |  | .954 | .961 | .953 |
|  |  |  |  | ${\bar{\hat{\beta}}}_{3,\mathrm{SP}}$ |  | .954 | .961 | .953 |
|  |  | Log Normal | MCAR | ${\bar{\hat{\beta}}}_{3,\mathrm{PS}}$ | .951 | .949 | .952 | .951 |
|  |  |  |  | ${\bar{\hat{\beta}}}_{3,\mathrm{SP}}$ |  | .949 | .950 | .949 |
|  |  |  | MAR | ${\bar{\hat{\beta}}}_{3,\mathrm{PS}}$ |  | .953 | .949 | .954 |
|  |  |  |  | ${\bar{\hat{\beta}}}_{3,\mathrm{SP}}$ |  | .953 | .949 | .954 |
| 0.45 | 100 | Normal | MCAR | ${\bar{\hat{\beta}}}_{3,\mathrm{PS}}$ | .947 | .964 | .959 | .961 |
|  |  |  |  | ${\bar{\hat{\beta}}}_{3,\mathrm{SP}}$ |  | .948 | .941 | .947 |
|  |  |  | MAR | ${\bar{\hat{\beta}}}_{3,\mathrm{PS}}$ |  | .960 | .956 | .962 |
|  |  |  |  | ${\bar{\hat{\beta}}}_{3,\mathrm{SP}}$ |  | .950 | .945 | .954 |
|  |  | Log Normal | MCAR | ${\bar{\hat{\beta}}}_{3,\mathrm{PS}}$ | .933 | .959 | .964 | .962 |
|  |  |  |  | ${\bar{\hat{\beta}}}_{3,\mathrm{SP}}$ |  | .948 | .950 | .954 |
|  |  |  | MAR | ${\bar{\hat{\beta}}}_{3,\mathrm{PS}}$ |  | .957 | .950 | .952 |
|  |  |  |  | ${\bar{\hat{\beta}}}_{3,\mathrm{SP}}$ |  | .944 | .947 | .940 |
|  | 500 | Normal | MCAR | ${\bar{\hat{\beta}}}_{3,\mathrm{PS}}$ | .956 | .959 | .960 | .959 |
|  |  |  |  | ${\bar{\hat{\beta}}}_{3,\mathrm{SP}}$ |  | .955 | .959 | .951 |
|  |  |  | MAR | ${\bar{\hat{\beta}}}_{3,\mathrm{PS}}$ |  | .964 | .971 | .962 |
|  |  |  |  | ${\bar{\hat{\beta}}}_{3,\mathrm{SP}}$ |  | .956 | .964 | .950 |
|  |  | Log Normal | MCAR | ${\bar{\hat{\beta}}}_{3,\mathrm{PS}}$ | .947 | .950 | .953 | .960 |
|  |  |  |  | ${\bar{\hat{\beta}}}_{3,\mathrm{SP}}$ |  | .938 | .942 | .955 |
|  |  |  | MAR | ${\bar{\hat{\beta}}}_{3,\mathrm{PS}}$ |  | .955 | .955 | .958 |
|  |  |  |  | ${\bar{\hat{\beta}}}_{3,\mathrm{SP}}$ |  | .945 | .949 | .954 |

Table 10: *Bias of standardized regression coefficient of predictor* $\mathbf{x}_{4}$ *(standard deviations between brackets). Entries must be multiplied by* 10^-3^.

| *ρ*² | *N* | Distr. | Miss.  Mech. | Comb.  Method | Percentage of missingness | | | |
| --- | --- | --- | --- | --- | --- | --- | --- | --- |
|  |  |  |  |  | 0% | 12.5% | 25% | 50% |
| 0 | 100 | Normal | MCAR | ${\bar{\hat{\beta}}}_{4,\mathrm{PS}}$ | 5 (116) | 3 (138) | 10 (161) | 1 (166) |
|  |  |  |  | ${\bar{\hat{\beta}}}_{4,\mathrm{SP}}$ |  | 3 (138) | 10 (161) | 1 (166) |
|  |  |  | MAR | ${\bar{\hat{\beta}}}_{4,\mathrm{PS}}$ |  | 4 (130) | 4 (130) | 1 (138) |
|  |  |  |  | ${\bar{\hat{\beta}}}_{4,\mathrm{SP}}$ |  | 4 (130) | 4 (130) | 1 (138) |
|  |  | Log  Normal | MCAR | ${\bar{\hat{\beta}}}_{4,\mathrm{PS}}$ | -4 (112) | -1 (130) | -10 (145) | -1 (151) |
|  |  |  |  | ${\bar{\hat{\beta}}}_{4,\mathrm{SP}}$ |  | -1 (130) | -10 (145) | -1 (151) |
|  |  |  | MAR | ${\bar{\hat{\beta}}}_{4,\mathrm{PS}}$ |  | -1 (114) | -4 (114) | -4 (114) |
|  |  |  |  | ${\bar{\hat{\beta}}}_{4,\mathrm{SP}}$ |  | -1 (114) | -4 (113) | -4 (114) |
|  | 500 | Normal | MCAR | ${\bar{\hat{\beta}}}_{4,\mathrm{PS}}$ | -3 (50) | -4 (59) | -2 (61) | -3 (62) |
|  |  |  |  | ${\bar{\hat{\beta}}}_{4,\mathrm{SP}}$ |  | -4 (59) | -2 (61) | -3 (62) |
|  |  |  | MAR | ${\bar{\hat{\beta}}}_{4,\mathrm{PS}}$ |  | -4 (54) | -3 (54) | -3 (55) |
|  |  |  |  | ${\bar{\hat{\beta}}}_{4,\mathrm{SP}}$ |  | -4 (54) | -3 (54) | -3 (55) |
|  |  | Log Normal | MCAR | ${\bar{\hat{\beta}}}_{4,\mathrm{PS}}$ | 2 (49) | 2 (62) | 1 (64) | 1 (66) |
|  |  |  |  | ${\bar{\hat{\beta}}}_{4,\mathrm{SP}}$ |  | 2 (62) | 1 (64) | 1 (66) |
|  |  |  | MAR | ${\bar{\hat{\beta}}}_{4,\mathrm{PS}}$ |  | 2 (49) | 2 (49) | 1 (49) |
|  |  |  |  | ${\bar{\hat{\beta}}}_{4,\mathrm{SP}}$ |  | 2 (49) | 2 (49) | 1 (49) |
| 0.45 | 100 | Normal | MCAR | ${\bar{\hat{\beta}}}_{4,\mathrm{PS}}$ | 5 (85) | 2 (105) | 3 (121) | -2 (125) |
|  |  |  |  | ${\bar{\hat{\beta}}}_{4,\mathrm{SP}}$ |  | 2 (105) | 3 (121) | -3 (125) |
|  |  |  | MAR | ${\bar{\hat{\beta}}}_{4,\mathrm{PS}}$ |  | 4 (96) | 2 (96) | 0 (103) |
|  |  |  |  | ${\bar{\hat{\beta}}}_{4,\mathrm{SP}}$ |  | 4 (96) | 2 (96) | 0 (103) |
|  |  | Log Normal | MCAR | ${\bar{\hat{\beta}}}_{4,\mathrm{PS}}$ | 0 (83) | -2 (99) | -15 (116) | -1 (123) |
|  |  |  |  | ${\bar{\hat{\beta}}}_{4,\mathrm{SP}}$ |  | -2 (99) | -15 (116) | -1 (123) |
|  |  |  | MAR | ${\bar{\hat{\beta}}}_{4,\mathrm{PS}}$ |  | 1 (90) | 1 (90) | 1 (91) |
|  |  |  |  | ${\bar{\hat{\beta}}}_{4,\mathrm{SP}}$ |  | 1 (90) | 1 (90) | 1 (90) |
|  | 500 | Normal | MCAR | ${\bar{\hat{\beta}}}_{4,\mathrm{PS}}$ | -2 (36) | -3 (44) | -1 (45) | -2 (45) |
|  |  |  |  | ${\bar{\hat{\beta}}}_{4,\mathrm{SP}}$ |  | -3 (44) | -1 (45) | -2 (45) |
|  |  |  | MAR | ${\bar{\hat{\beta}}}_{4,\mathrm{PS}}$ |  | -2 (39) | -2 (39) | -2 (40) |
|  |  |  |  | ${\bar{\hat{\beta}}}_{4,\mathrm{SP}}$ |  | -3 (39) | -2 (39) | -2 (40) |
|  |  | Log Normal | MCAR | ${\bar{\hat{\beta}}}_{4,\mathrm{PS}}$ | 3 (36) | 6 (42) | 5 (43) | 6 (45) |
|  |  |  |  | ${\bar{\hat{\beta}}}_{4,\mathrm{SP}}$ |  | 6 (42) | 5 (43) | 6 (45) |
|  |  |  | MAR | ${\bar{\hat{\beta}}}_{4,\mathrm{PS}}$ |  | 5 (37) | 4 (37) | 4 (37) |
|  |  |  |  | ${\bar{\hat{\beta}}}_{4,\mathrm{SP}}$ |  | 5 (37) | 4 (37) | 4 (37) |

Table 11: *Results coverage percentages of standardized regression coefficient of predictor* $\mathbf{x}_{4}$.

| *ρ*² | *N* | Distribution | Missingness  Mechanism | Combination Method | Percentage of missingness | | | |
| --- | --- | --- | --- | --- | --- | --- | --- | --- |
|  |  |  |  |  | 0% | 12.5% | 25% | 50% |
| 0 | 100 | Normal | MCAR | ${\bar{\hat{\beta}}}_{4,\mathrm{PS}}$ | .942 | .936 | .945 | .954 |
|  |  |  |  | ${\bar{\hat{\beta}}}_{4,\mathrm{SP}}$ |  | .937 | .943 | .951 |
|  |  |  | MAR | ${\bar{\hat{\beta}}}_{4,\mathrm{PS}}$ |  | .941 | .957 | .945 |
|  |  |  |  | ${\bar{\hat{\beta}}}_{4,\mathrm{SP}}$ |  | .941 | .956 | .945 |
|  |  | Log Normal | MCAR | ${\bar{\hat{\beta}}}_{4,\mathrm{PS}}$ | .950 | .953 | .944 | .953 |
|  |  |  |  | ${\bar{\hat{\beta}}}_{4,\mathrm{SP}}$ |  | .953 | .944 | .954 |
|  |  |  | MAR | ${\bar{\hat{\beta}}}_{4,\mathrm{PS}}$ |  | .952 | .955 | .948 |
|  |  |  |  | ${\bar{\hat{\beta}}}_{4,\mathrm{SP}}$ |  | .953 | .952 | .949 |
|  | 500 | Normal | MCAR | ${\bar{\hat{\beta}}}_{4,\mathrm{PS}}$ | .949 | .948 | .963 | .951 |
|  |  |  |  | ${\bar{\hat{\beta}}}_{4,\mathrm{SP}}$ |  | .948 | .963 | .951 |
|  |  |  | MAR | ${\bar{\hat{\beta}}}_{4,\mathrm{PS}}$ |  | .946 | .955 | .951 |
|  |  |  |  | ${\bar{\hat{\beta}}}_{4,\mathrm{SP}}$ |  | .947 | .955 | .951 |
|  |  | Log Normal | MCAR | ${\bar{\hat{\beta}}}_{4,\mathrm{PS}}$ | .948 | .937 | .931 | .921 |
|  |  |  |  | ${\bar{\hat{\beta}}}_{4,\mathrm{SP}}$ |  | .937 | .931 | .920 |
|  |  |  | MAR | ${\bar{\hat{\beta}}}_{4,\mathrm{PS}}$ |  | .955 | .950 | .952 |
|  |  |  |  | ${\bar{\hat{\beta}}}_{4,\mathrm{SP}}$ |  | .955 | .950 | .952 |
| 0.45 | 100 | Normal | MCAR | ${\bar{\hat{\beta}}}_{4,\mathrm{PS}}$ | .945 | .941 | .957 | .960 |
|  |  |  |  | ${\bar{\hat{\beta}}}_{4,\mathrm{SP}}$ |  | .934 | .953 | .958 |
|  |  |  | MAR | ${\bar{\hat{\beta}}}_{4,\mathrm{PS}}$ |  | .950 | .958 | .952 |
|  |  |  |  | ${\bar{\hat{\beta}}}_{4,\mathrm{SP}}$ |  | .944 | .952 | .950 |
|  |  | Log Normal | MCAR | ${\bar{\hat{\beta}}}_{4,\mathrm{PS}}$ | .955 | .950 | .957 | .953 |
|  |  |  |  | ${\bar{\hat{\beta}}}_{4,\mathrm{SP}}$ |  | .949 | .955 | .949 |
|  |  |  | MAR | ${\bar{\hat{\beta}}}_{4,\mathrm{PS}}$ |  | .951 | .953 | .945 |
|  |  |  |  | ${\bar{\hat{\beta}}}_{4,\mathrm{SP}}$ |  | .950 | .950 | .944 |
|  | 500 | Normal | MCAR | ${\bar{\hat{\beta}}}_{4,\mathrm{PS}}$ | .955 | .945 | .971 | .956 |
|  |  |  |  | ${\bar{\hat{\beta}}}_{4,\mathrm{SP}}$ |  | .945 | .970 | .956 |
|  |  |  | MAR | ${\bar{\hat{\beta}}}_{4,\mathrm{PS}}$ |  | .953 | .953 | .954 |
|  |  |  |  | ${\bar{\hat{\beta}}}_{4,\mathrm{SP}}$ |  | .949 | .952 | .955 |
|  |  | Log Normal | MCAR | ${\bar{\hat{\beta}}}_{4,\mathrm{PS}}$ | .944 | .955 | .951 | .945 |
|  |  |  |  | ${\bar{\hat{\beta}}}_{4,\mathrm{SP}}$ |  | .950 | .949 | .942 |
|  |  |  | MAR | ${\bar{\hat{\beta}}}_{4,\mathrm{PS}}$ |  | .943 | .954 | .946 |
|  |  |  |  | ${\bar{\hat{\beta}}}_{4,\mathrm{SP}}$ |  | .940 | .952 | .944 |

Table 12: *Bias of* $R^{2}$ *for methods* $\hat{R}_{\mathrm{PS}}^{2}$ *and* $\hat{R}_{\mathrm{SP}}^{2}$ *(standard deviations between brackets). Entries must be multiplied by* 10^-3^.

| *ρ*² | *N* | Distr. | Miss.  Mech. | Comb.  Method | Percentage of missingness | | | |
| --- | --- | --- | --- | --- | --- | --- | --- | --- |
|  |  |  |  |  | 0% | 12.5% | 25% | 50% |
| 0 | 100 | Normal | MCAR | $\hat{R}_{\mathrm{PS}}^{2}$ | 42 (28) | 57 (38) | 75 (52) | 78 (58) |
|  |  |  |  | $\hat{R}_{\mathrm{SP}}^{2}$ |  | 57 (38) | 75 (52) | 77 (58) |
|  |  |  | MAR | $\hat{R}_{\mathrm{PS}}^{2}$ |  | 53 (35) | 55 (37) | 57 (41) |
|  |  |  |  | $\hat{R}_{\mathrm{SP}}^{2}$ |  | 53 (35) | 55 (37) | 57 (41) |
|  |  | Log  Normal | MCAR | $\hat{R}_{\mathrm{PS}}^{2}$ | 42 (28) | 52 (36) | 62 (45) | 65 (48) |
|  |  |  |  | $\hat{R}_{\mathrm{SP}}^{2}$ |  | 52 (36) | 62 (45) | 64 (48) |
|  |  |  | MAR | $\hat{R}_{\mathrm{PS}}^{2}$ |  | 44 (30) | 46 (31) | 45 (31) |
|  |  |  |  | $\hat{R}_{\mathrm{SP}}^{2}$ |  | 44 (30) | 46 (31) | 45 (31) |
|  | 500 | Normal | MCAR | $\hat{R}_{\mathrm{PS}}^{2}$ | 8 (6) | 11 (8) | 12 (8) | 12 (9) |
|  |  |  |  | $\hat{R}_{\mathrm{SP}}^{2}$ |  | 11 (8) | 12 (8) | 12 (9) |
|  |  |  | MAR | $\hat{R}_{\mathrm{PS}}^{2}$ |  | 9 (7) | 9 (6) | 10 (7) |
|  |  |  |  | $\hat{R}_{\mathrm{SP}}^{2}$ |  | 9 (7) | 9 (6) | 10 (7) |
|  |  | Log Normal | MCAR | $\hat{R}_{\mathrm{PS}}^{2}$ | 8 (6) | 11 (8) | 12 (8) | 12 (9) |
|  |  |  |  | $\hat{R}_{\mathrm{SP}}^{2}$ |  | 11 (8) | 12 (8) | 12 (9) |
|  |  |  | MAR | $\hat{R}_{\mathrm{PS}}^{2}$ |  | 9 (6) | 9 (6) | 8 (6) |
|  |  |  |  | $\hat{R}_{\mathrm{SP}}^{2}$ |  | 9 (6) | 9 (6) | 8 (6) |
| 0.45 | 100 | Normal | MCAR | $\hat{R}_{\mathrm{PS}}^{2}$ | 20 (71) | 21 (84) | 25 (98) | 23 (99) |
|  |  |  |  | $\hat{R}_{\mathrm{SP}}^{2}$ |  | 21 (84) | 23 (98) | 21 (98) |
|  |  |  | MAR | $\hat{R}_{\mathrm{PS}}^{2}$ |  | 19 (80) | 18 (84) | 19 (84) |
|  |  |  |  | $\hat{R}_{\mathrm{SP}}^{2}$ |  | 18 (80) | 17 (84) | 19 (84) |
|  |  | Log Normal | MCAR | $\hat{R}_{\mathrm{PS}}^{2}$ | 12 (80) | 3 (93) | -13 (104) | -15 (106) |
|  |  |  |  | $\hat{R}_{\mathrm{SP}}^{2}$ |  | 2 (93) | -14 (104) | -16 (106) |
|  |  |  | MAR | $\hat{R}_{\mathrm{PS}}^{2}$ |  | -6 (81) | -7 (83) | -8 (84) |
|  |  |  |  | $\hat{R}_{\mathrm{SP}}^{2}$ |  | -7 (81) | -7 (83) | -8 (84) |
|  | 500 | Normal | MCAR | $\hat{R}_{\mathrm{PS}}^{2}$ | 3 (33) | 3 (38) | 3 (39) | 3 (39) |
|  |  |  |  | $\hat{R}_{\mathrm{SP}}^{2}$ |  | 2 (38) | 3 (39) | 3 (39) |
|  |  |  | MAR | $\hat{R}_{\mathrm{PS}}^{2}$ |  | 3 (35) | 3 (35) | 3 (35) |
|  |  |  |  | $\hat{R}_{\mathrm{SP}}^{2}$ |  | 3 (35) | 3 (35) | 3 (35) |
|  |  | Log Normal | MCAR | $\hat{R}_{\mathrm{PS}}^{2}$ | -2 (37) | -4 (42) | -5 (43) | -4 (43) |
|  |  |  |  | $\hat{R}_{\mathrm{SP}}^{2}$ |  | -4 (42) | -5 (43) | -4 (43) |
|  |  |  | MAR | $\hat{R}_{\mathrm{PS}}^{2}$ |  | -8 (37) | -8 (37) | -8 (37) |
|  |  |  |  | $\hat{R}_{\mathrm{SP}}^{2}$ |  | -9 (37) | -8 (37) | -8 (37) |

Table 13: *Bias of* $R^{2}$ *for methods* $\mathfrak{R}^{2}$ *and* $\bar{R^{2}}$ *(standard deviations between brackets). Entries must be multiplied by* 10^-3^.

| *ρ*² | *N* | Distr. | Miss.  Mech. | Comb.  Method | Percentage of missingness | | | |
| --- | --- | --- | --- | --- | --- | --- | --- | --- |
|  |  |  |  |  | 0% | 12.5% | 25% | 50% |
| 0 | 100 | Normal | MCAR | $\bar{R^{2}}$ | 42 (28) | 74 (38) | 109 (54) | 117 (62) |
|  |  |  |  | $\mathfrak{R}^{2}$ |  | 70 (38) | 102 (54) | 110 (63) |
|  |  |  | MAR | $\bar{R^{2}}$ |  | 66 (35) | 71 (39) | 74 (43) |
|  |  |  |  | $\mathfrak{R}^{2}$ |  | 63 (35) | 68 (39) | 70 (43) |
|  |  | Log  Normal | MCAR | $\bar{R^{2}}$ | 42 (28) | 67 (36) | 91 (46) | 96 (51) |
|  |  |  |  | $\mathfrak{R}^{2}$ |  | 63 (36) | 85 (46) | 90 (52) |
|  |  |  | MAR | $\bar{R^{2}}$ |  | 53 (31) | 54 (32) | 53 (31) |
|  |  |  |  | $\mathfrak{R}^{2}$ |  | 50 (31) | 52 (32) | 51 (32) |
|  | 500 | Normal | MCAR | $\bar{R^{2}}$ | 8 (6) | 14 (8) | 16 (9) | 17 (9) |
|  |  |  |  | $\mathfrak{R}^{2}$ |  | 13 (8) | 15 (9) | 15 (9) |
|  |  |  | MAR | $\bar{R^{2}}$ |  | 11 (7) | 11 (7) | 11 (7) |
|  |  |  |  | $\mathfrak{R}^{2}$ |  | 10 (7) | 10 (7) | 11 (7) |
|  |  | Log Normal | MCAR | $\bar{R^{2}}$ | 8 (6) | 14 (8) | 16 (9) | 17 (9) |
|  |  |  |  | $\mathfrak{R}^{2}$ |  | 13 (8) | 15 (9) | 15 (9) |
|  |  |  | MAR | $\bar{R^{2}}$ |  | 10 (6) | 10 (6) | 10 (6) |
|  |  |  |  | $\mathfrak{R}^{2}$ |  | 9 (6) | 9 (6) | 9 (6) |
| 0.45 | 100 | Normal | MCAR | $\bar{R^{2}}$ | 20 (71) | 47 (82) | 59 (95) | 61 (94) |
|  |  |  |  | $\mathfrak{R}^{2}$ |  | 42 (82) | 55 (94) | 56 (94) |
|  |  |  | MAR | $\bar{R^{2}}$ |  | 43 (79) | 44 (83) | 46 (83) |
|  |  |  |  | $\mathfrak{R}^{2}$ |  | 38 (79) | 39 (82) | 40 (82) |
|  |  | Log Normal | MCAR | $\bar{R^{2}}$ | 12 (80) | 28 (92) | 19 (101) | 20 (101) |
|  |  |  |  | $\mathfrak{R}^{2}$ |  | 22 (91) | 15 (101) | 15 (102) |
|  |  |  | MAR | $\bar{R^{2}}$ |  | 17 (80) | 17 (82) | 15 (83) |
|  |  |  |  | $\mathfrak{R}^{2}$ |  | 12 (80) | 11 (81) | 10 (82) |
|  | 500 | Normal | MCAR | $\bar{R^{2}}$ | 3 (33) | 22 (38) | 24 (39) | 23 (39) |
|  |  |  |  | $\mathfrak{R}^{2}$ |  | 17 (38) | 19 (39) | 18 (39) |
|  |  |  | MAR | $\bar{R^{2}}$ |  | 22 (35) | 22 (35) | 23 (35) |
|  |  |  |  | $\mathfrak{R}^{2}$ |  | 17 (35) | 17 (35) | 18 (35) |
|  |  | Log Normal | MCAR | $\bar{R^{2}}$ | -2 (37) | 16 (42) | 15 (43) | 16 (43) |
|  |  |  |  | $\mathfrak{R}^{2}$ |  | 11 (41) | 10 (43) | 11 (43) |
|  |  |  | MAR | $\bar{R^{2}}$ |  | 10 (37) | 10 (37) | 11 (37) |
|  |  |  |  | $\mathfrak{R}^{2}$ |  | 5 (37) | 5 (37) | 6 (37) |
